# Simulation of emulsion evolution in shear flow field under the control of bidirectional pulsed electric field

**DOI:** 10.1371/journal.pone.0336345

**Published:** 2026-07-10

**Authors:** Heping Wang, Yi Wu, Yijian Peng, Xiaohang Zhang

**Affiliations:** Key Laboratory of Engineering Mathematics and Advanced Computing of Jiangxi University of Water Resources and Electric Power, School of Sciences, Jiangxi University of Water Resources and Electric Power, Nanchang, People’s Republic of China; University of Salento, ITALY

## Abstract

Electrostatic demulsification is a widely used technique for treating high-water-cut emulsions (O/W type). However, in high-water-cut emulsions, continuous electric fields often lead to short-circuiting and chain formation. Meanwhile, pure shear fields rely on random collisions, which is inefficient for fine droplets. To address these limitations, this article established a lattice Boltzmann model coupled with electric and shear field. It explored the impact of the combined action of bidirectional pulsed electric field (BPEF) and shear field on the demulsification process. Simulation results reveal that: Under the action of pure shear field, increasing the shear effect will lead oil droplets to move around the flow field center, and forming a stable state eventually. However, after applying bidirectional pulsed electric field on both sides of the flow field, the electric field force breaks this stable state and promotes further aggregation of oil droplets. Analysis of streamline diagrams indicates that: Bidirectional pulsed electric field periodically changes the direction of the electric field force. It leads to induce an unbalanced velocity distribution in the flow field. And this phenomenon can accelerate or decelerate the aggregation of oil droplets. Quantitative analysis indicates that the synergistic coupling of BPEF and shear field significantly outperforms single-field methods. Under the optimal electric potential of V = 300 and shear rate of γ=0.001, the composite morpho-dynamic index ($D_{r}$) reached 11.5. This represents a 4.7-fold increase compared to the pure shear field ($D_{r}\approx 2.0$) and a 109% improvement compared to the low-voltage condition (V = 100), indicating a highly aggregated and moderately deformed stable state. These simulation results are significant important for understand the synergistic demulsification effects of electric and shear fields.

## 1. Introduction

With the rapid development of industrial production, efficient demulsification of emulsions has become an important research topic [1–3]. Such as oilfield development and chemical production in fields, the stable state of emulsions poses a significant challenge to oil-water separation [4,5]. Traditional demulsification methods are often inefficient and may cause secondary environmental pollution [6, 7–9]. Therefore, exploring new demulsification technologies has significant practical significance and application value [10–12].

Bidirectional pulsed electric fields have been proven to be an efficient demulsification method [13,14]. In terms of experiments, some scholars have studied the demulsification effects of bidirectional pulsed electric fields on water-in-oil emulsions, investigating the impact of voltage, frequency, duty cycle, and emulsion conductivity on the demulsification of oil-water emulsions [15]. Other studies have discovered the chaining and aggregation behavior of oil droplets in oil-water emulsions under the influence of bidirectional direct current pulsed electric fields, demonstrating that the demulsification performance of bidirectional pulsed electric fields is significantly better than that of direct current or alternating current electric fields [16]. In terms of numerical simulation, some studies have simulated the dynamic response of droplets in low-viscosity fluids under pulsed direct current and sinusoidal electric fields [17]. Additionally, some scholars have established mathematical models to simulate the impact of the coupling of electric and flow fields on the separation of O/W emulsions [18]. Concurrently, some studies have used molecular dynamics simulations to investigate the effect of direct current electric fields on the movement of oil droplets, revealing negative electrophoretic migration [19]. This method has also been applied to study the influence of bidirectional pulsed electric fields on the transformation and aggregation behavior of oil droplets in emulsions. Simulation results have confirmed the deformation and movement of oil droplets in the direction of the electric field [20]. However, the action of a single electric field often fails to achieve complete demulsification, especially when dealing with highly stable emulsions. Therefore, combining other physical fields such as shear fields to enhance demulsification effects has become a new direction for research [21].

As a common type of fluid mechanical field, shear fields can disrupt the stability of droplets in emulsions by applying shear forces. When coupled with electric fields, a synergistic effect can be produced, further enhancing demulsification efficiency [22,23]. In terms of experiments, some scholars have studied the impact of the combined action of direct current electric fields and shear flow fields on droplet deformation through visualization experiments [24]. Some studies have revealed the interactions between electric field forces, viscous forces, and interfacial tension during the droplet fragmentation process, which is under the combined action of electric and shear flow fields [25]. In terms of numerical simulation, some researchers have reported in detail the LBM (lattice Boltzmann method) model for studying multiphase fluids [26]. Some researchers have proposed a new phase-field-based lattice Boltzmann method for the dynamic flow of axisymmetric two-phase current. The method consists of three LB models, which are used to solve the axisymmetric Allen-Cahn equation of the phase field, the axisymmetric Poisson equation of the potential and the axisymmetric Navier-Stokes equation of the flow field, respectively [27]. Some scholars have found that changing the dielectric constant and conductivity can regulate droplet deformation in shear flow fields, by solving two-dimensional current fluid dynamics equations and using the fluid volume method [28]. Another study used molecular dynamics methods to investigate the effect of flow field shear on the electrocoalescence of water droplets in oil-in-water emulsions, revealing the relationship between shear rate and droplet angle on droplet coalescence. However, the mechanism of demulsification by the coupled action of bidirectional pulsed electric fields and shear fields remains insufficiently studied.

This study aims to explore the technical principles and application effects of demulsification by coupling bidirectional pulsed electric fields with shear fields. In this paper, a mathematical model combining pulsed electric fields and shear fields is established by using the lattice Boltzmann method. And the evolution of emulsions under shear flow without electric fields, different shear rates, different electric field directions (perpendicular or parallel to the shear direction), and different electric field intensities is simulated. Moreover, the streamlines of the flow field are used to analyze how bidirectional triangular pulsed electric fields affect the aggregation of oil droplet by changing the flow field distribution. Additionally, a quantitative analysis of the impact of different conditions on the demulsification is conducted. The research findings of this paper can provide theoretical basis and technical support for the treatment of industrial emulsions, in order to achieve more efficient and environmentally friendly demulsification treatment.

## 2. Numerical method

### 2.1. Lattice Boltzmann method

In this study, all governing equations are formulated within the lattice Boltzmann framework. Unless otherwise specified, all variables are expressed in dimensionless lattice units. The corresponding physical meanings and SI units are summarized in the Symbol Table 1. A dimensional mapping between lattice units and physical quantities is provided in Section 3 to facilitate physical interpretation.

**Table 1:** Symbol description list.

Symbol	Physical meaning	Type	Unit (SI)
ρ	Density	Scalar	${\text{kg/m}}^{\text{3}}$
φ	Electric potential	Scalar	V
ε	Permittivity	Scalar	F/m
σ	Interfacial tension	Scalar	N/m
μ	Dynamic viscosity	Scalar	Pa·s
γ	Shear rate	Scalar	${\text{s}}^{-1}$
V	Dimensionless voltage	Scalar	Dimensionless (—)
Dr	Aggregation degree	Scalar	Dimensionless (—)
A	Droplet area	Scalar	${\text{m}}^{\text{2}}$
P	Droplet perimeter	Scalar	m
t	Time	Scalar	s
u	Velocity	Vector	m/s
E	Electric field	Vector	V/m
F	Total body force	Vector	${\text{N/m}}^{\text{3}}$
$F_{e}$	Electric field force	Vector	${\text{N/m}}^{\text{3}}$
$F_{s}$	Surface tension force	Vector	${\text{N/m}}^{\text{3}}$
∇	Gradient operator	Vector operator	${\text{m}}^{-1}$

Note: All variables in the numerical simulation are expressed in dimensionless lattice Boltzmann units (lattice units, lu and time steps, ts) unless otherwise specified. The SI units listed here correspond to their physical interpretations used for dimensional mapping.

Due to the periodic change of the electric field, the electromagnetic induction effect will produce a magnetic field. However, in this paper, the current generated by the internal charge flow of the fluid is very small, so the influence of the magnetic field force is ignored. For the calculation of electric field distribution in the flow field, the central difference method is used in this paper. The electric potential distribution φ within the computational domain is governed by the Poisson equation for a heterogeneous dielectric medium [29]:


$$\begin{array}{c} \nabla \cdot [\varepsilon_{b}(x)\nabla \varphi ]=-\rho_{e} \end{array}$$ (1)

Among them, φ is the potential, $\rho_{e}$ is the charge density and $\varepsilon_{b}$ is the dielectric constant of water. In order to describe the distribution of the electric field in the medium, the relationship between the electric field and the electric potential is:


$$\begin{array}{c} E=-\nabla \varphi \end{array}$$ (2)

Using Maxwell stress tensor to represent the electric field force on the droplet can obtain:


$$\begin{array}{c} F_{E}=\nabla \left(\varepsilon_{\text{b}}\varepsilon_{\text{r}}EE\right)-\frac{\text{1}}{\text{2}}E^{\text{2}}\nabla \varepsilon_{\text{b}}\varepsilon_{\text{r}} \end{array}$$ (3)

The calculation area is divided into grids, and the numerical solution of the electric potential is calculated at each grid point. After discretization, the grid spacing in the x and y directions is Δx and Δy, and the grid point coordinate is $\left(x_{i,}y_{i}\right)$. The central difference method is used to discretize the Poisson equation, and the difference of the second derivative is approximated as:


$$\begin{array}{c} \nabla^{\text{2}}\varphi \approx \frac{\left(\varphi_{i+1,j}-2\varphi_{i,j}+\varphi_{i-1,j}\right)}{\Delta x^{\text{2}}}+\frac{\left(\varphi_{i,j+1}-2\varphi_{i,j}+\varphi_{i,j-1}\right)}{\Delta y^{\text{2}}} \end{array}$$ (4)

The Gauss-Seidel iteration method is used to gradually update the potential value of each grid point until the convergence condition is satisfied. The update formula of Gauss-Seidel iterative method is:


$$\begin{array}{c} \overset{k+1}{\underset{i,j}{\varphi }}=\frac{\text{1}}{\text{4}}\left[\frac{\left(\overset{k}{\underset{i+1,j}{\varphi }}+\overset{k}{\underset{i-1,j}{\phi }}\right)}{\Delta x^{\text{2}}}+\frac{\left(\overset{k}{\underset{i,j+1}{\varphi }}+\overset{k}{\underset{i,j-1}{\varphi }}\right)}{\Delta y^{\text{2}}}+E_{\rho }\right] \end{array}$$ (5)

Here, $\overset{k}{\underset{i,j}{\varphi }}$ represents the potential value of grid point (i,j) at the k iteration, and $E_{\rho }$ is a term related to the charge distribution. For the criteria of stopping iteration, there are: $max\left|\overset{k+1}{\underset{i,j}{\phi }}-\overset{k}{\underset{i,j}{\phi }}\right|<\varepsilon_{abs}$, the value of $\varepsilon_{abs}$ is $10^{-6}$.

Since the electric field is a negative gradient of the potential, the electric field calculated by the differential approximation is:


$$\begin{array}{c} E_{x}\approx -\frac{\varphi_{i+1,j}-\varphi_{i-1,j}}{\Delta x} \end{array}$$ (6)

$$\begin{array}{c} E_{y}\approx -\frac{\varphi_{i,j+1}-\varphi_{i,j-1}}{\Delta y} \end{array}$$ (7)

Finally, by calculating the electric field components $E_{x}$ and $E_{y}$ of each grid point, the electric field distribution of the whole region can be obtained.

The lattice Boltzmann method is a discretized Boltzmann equation. In this paper, the two-dimensional model of D2Q9 is used. The distribution function in the model contains nine directions and is in the same two-dimensional plane. The lattice vector is represented by $c_{i}$, and there are


$$\begin{array}{c} c_{i}=\left\{ \begin{array}{cc} @lll@\text{(}0,0\text{)} & i=0\\ c(\text{cos}((i-1)\frac{\pi }{\text{2}}),\text{sin}((i-1)\frac{\pi }{\text{2}})) & i=1,2,3,4\\ \sqrt{\text{2}}c\left(\text{cos}((2i-1)\frac{\pi }{\text{4}}),\text{sin}\left((2i-1)\frac{\pi }{\text{4}}\right)\right) & i=5,6,7,8 \end{array}\right. \end{array}$$ (8)

The evolution equation of distribution function after discretization of particle motion is:


$$\begin{array}{c} f_{i}(x+c_{i}\delta_{t},t+\delta_{t})-f_{i}\left(x,t\right)=\Omega_{ki}\left(\overset{eq}{\underset{i}{f}}\left(x,t\right)\right) \end{array}$$ (9)

Where $f_{i}\left(x,t\right)$ is the discrete velocity of time t and position x, $\delta_{t}$ is the time step, and $\Omega_{ki}\left(\overset{eq}{\underset{i}{f}}\left(x,t\right)\right)$ is the collision term. After the BGK (Bhatnagar-Gross-Krook) approximation of the LBM equation, the collision term can be expressed as:


$$\begin{array}{c} \Omega_{ki}\left(\overset{eq}{\underset{i}{f}}\left(x,t\right)\right)=-\frac{\text{1}}{\tau }\left(f_{i}\left(x,t)-\overset{eq}{\underset{i}{f}}(x,t\right)\right) \end{array}$$ (10)

Therefore, the LBM control equation in the form of BGK can be obtained [30]:


$$\begin{array}{c} f_{i}(x+c_{i}\delta_{t},t+\delta_{t})-f_{i}\left(x,t\right)=-\frac{\text{1}}{\tau }\left(f_{i}\left(x,t)-\overset{eq}{\underset{i}{f}}(x,t\right)\right) \end{array}$$ (11)

Where τ is the relaxation factor, which represents the time required for the distribution function to reach equilibrium.

The differential form of the governing equation can be expressed as:


$$\begin{array}{c} \rho \frac{\partial v}{\partial t}+\rho (u\cdot \nabla )=\nabla \cdot (-pI+\tau )+F_{st}+F_{E} \end{array}$$ (12)

Where ρ, u, p, I, $F_{st}$ and $F_{E}$ are fluid density, fluid velocity, pressure, unit matrix, viscous shear stress tensor, interfacial tension and electric field force, respectively.

Since the fluid studied in this paper is isothermal and weakly compressible, therefore:


$$\begin{array}{c} \nabla \cdot v=0 \end{array}$$ (13)

$\overset{eq}{\underset{i}{f}}\left(x,t\right)$ is the distribution function when the particle motion reaches equilibrium:


$$\begin{array}{c} \overset{eq}{\underset{i}{f}}=\omega_{i}\rho (1+\frac{u}{\overset{\text{2}}{\underset{s}{c}}}+\frac{u^{\text{2}}}{2{c_{s}}^{\text{4}}}-\frac{u^{\text{2}}}{2{c_{s}}^{\text{2}}}) \end{array}$$ (14)

Among them, u is the macro speed, $c_{s}$ is the speed of sound in a lattice. And $\omega_{i}$ as a weight coefficient can be expressed as


$$\begin{array}{c} \omega_{i}=\left\{ \begin{array}{cc} @ll@4/9 & i=1\\ 1/9 & i=2,3,4,5\\ 1/36 & i=6,7,8,9 \end{array}\right. \end{array}$$ (15)

To isolate the synergistic mechanism of the bidirectional pulsed electric field (BPEF) and the shear field, this study assumes an isothermal environment and neglects the effect of gravity.

For micro-scale emulsions (with typical droplet sizes of 10 100μm), the relative importance of gravity compared to interfacial tension is characterized by the Bond number ($Bo=\Delta \rho gR^{\text{2}}/\sigma$). Given the micro-scale dimensions and fluid properties, the Bond number is much smaller than unity (Bo≪1), indicating that the droplet dynamics are completely dominated by the Maxwell stress, viscous shear, and interfacial capillary forces, making gravity negligible.

Furthermore, the isothermal assumption is adopted to deliberately exclude the complex interference of thermocapillary (Marangoni) flows and temperature-dependent viscosity variations, thereby allowing a focused investigation into the pure electro-hydrodynamic (EHD) coupling laws.

### 2.2. The color model of the phase-separating lattice Boltzmann method

This study employs the LBM color model. Initially proposed by Gunstensen [31], this model is designed to simulate the separation process of different fluids. In this model, different fluids are assigned distinct colors. The model uses independent distribution functions to describe the evolution of particles within the fluid. Meanwhile, Grunau, Eggert and Chen [32] introduced the concept of color gradient, which can be used to represent the interactions between different fluids. However, the issue of miscibility between different fluids still needs to be addressed. Therefore, some scholars [33,34] have proposed a recoloring method. This method ensures the isotropy of the phase interface and also resolves the problem of excessively high spurious velocities at the phase interface.

In this paper, water is the continuous phase, and oil droplets are the dispersed phase. They are represented by blue and red, respectively. The subscripts are “*b*” and “*r*,” respectively. The collision operator $\Omega_{ki}\left(x,t\right)$ can be represented by three different operators:


$$\begin{array}{c} \Omega_{ki}\left(x,t\right)=\overset{\text{3}}{\underset{ki}{\Omega }}\left(x,t\right)\left(\overset{\text{1}}{\underset{ki}{\Omega }}\left(x,t)+\overset{\text{2}}{\underset{ki}{\Omega }}(x,t\right)\right) \end{array}$$ (16)

Here, the symbol ‘ *k* ’ indicates either ‘ *b* ’ or ‘ *r* ’. $\overset{\text{1}}{\underset{ki}{\Omega }}\left(x,t\right)$ is the single-phase collision operator. It can describe the mutual collision behavior of particles within the fluid. This operator is represented as:


$$\begin{array}{c} \overset{\text{1}}{\underset{ki}{\Omega }}\left(x,t\right)=f_{ki}\left(x,t\right)-\tau \left(f_{ki}\left(x,t)-\overset{\text{(eq)}}{\underset{ki}{f}}(x,t\right)\right) \end{array}$$ (17)

Here, τ represents the relaxation factor, and $\overset{\text{(eq)}}{\underset{ki}{f}}$ is the equilibrium distribution function, denoted as:


$$\begin{array}{c} \overset{\;eq}{\underset{ki}{f}}=\rho_{k}\left(\overset{k}{\underset{i}{\varphi }}+\omega_{i}\left(1+\frac{u}{\overset{\text{2}}{\underset{s}{c}}}+\frac{u^{\text{2}}}{2\overset{\text{4}}{\underset{s}{c}}}-\frac{u^{\text{2}}}{2\overset{\text{2}}{\underset{s}{c}}}\right)\right) \end{array}$$ (18)

$\overset{k}{\underset{i}{\phi }}$ is a function of $\alpha_{k}$, and


$$\begin{array}{c} \overset{k}{\underset{i}{\phi }}=\left\{ \begin{array}{ccc} @ccc@\alpha_{k} & & \;\;\;\;i=1\\ (1-\alpha_{k})/5 & & i=2,3,4,5\\ (1-\alpha_{k})/20 & & i=6,7,8,9 \end{array}\right. \end{array}$$ (19)

$\alpha_{k}$ is a free parameter satisfying $\alpha_{k}\in \left(0,1\right)$.

The macroscopic density and velocity are:


$$\begin{array}{c} \rho_{k}=\sum_{i}f_{ki} \end{array}$$ (20)

$$\begin{array}{c} u=\frac{\text{1}}{\rho }\sum_{i}\sum_{k}f_{ki}c_{i} \end{array}$$ (21)

$\overset{\text{2}}{\underset{ki}{\Omega }}\left(x,t\right)$ represents the interface perturbation operator. It can represent the effect of surface tension on different fluids. The color gradient *F* at the interface can be expressed as


$$\begin{array}{c} F\left(x\right)=\sum_{\text{i}}c_{i}\left(\rho_{r}\left(x+c_{i})-\rho_{b}(x+c_{i}\right)\right) \end{array}$$ (22)

The perturbation operator satisfies the following equation:


$$\begin{array}{c} \overset{\text{2}}{\underset{ki}{\Omega }}(\text{x},\text{t)}=\frac{A_{K}}{\text{2}}\left|F\left(x\right)\right|(\omega_{i}\frac{(F\left(x\right)\cdot c_{i}{)}^{\text{2}}}{{\left|F\left(x\right)\right|}^{\text{2}}})-B_{i} \end{array}$$ (23)

$A_{k}$ is a free parameter to couple the red and blue phases, and and $\omega_{i}$ is a weight coefficient.


$$\begin{array}{c} B_{i}=\left\{ \begin{array}{cc} @ll@-4/27 & i=1\\ 2/27 & i=2,3,4,5\\ 5/108 & i=6,7,8,9 \end{array}\right. \end{array}$$ (24)

$\overset{\text{3}}{\underset{ki}{\Omega }}\left(x,t\right)$ represents the recoloring operator. It can prevent the miscibility of particles of different fluids. Some scholars[34] have proposed a simplified method. It can ensure that the multiphase fluids in the model are immiscible. The recoloring operator satisfies the following equation:


$$\begin{array}{c} \overset{\text{3}}{\underset{ri}{\Omega }}\left(f_{ri}\right)=\frac{\rho_{r}}{\rho }f_{i}+\beta \frac{\rho_{r}\rho_{b}}{\rho^{\text{2}}}\text{cos}\left(\theta_{i}\right)\sum_{k}\overset{e}{\underset{ki}{f}}\left(\rho_{k},0,\alpha_{k}\right) \end{array}$$ (25)

$$\begin{array}{c} \overset{\text{3}}{\underset{bi}{\Omega }}\left(f_{bi}\right)=\frac{\rho_{r}}{\rho }f_{i}-\beta \frac{\rho_{r}\rho_{b}}{\rho^{\text{2}}}\text{cos}\left(\theta_{i}\right)\sum_{k}\overset{e}{\underset{ki}{f}}\left(\rho_{k},0,\alpha_{k}\right) \end{array}$$ (26)

$\theta_{i}$ represents the angle between the direction vector and the color gradient unit vector.

To ensure phase separation and immiscibility, the segregation parameter β is set to 0.7 in the recoloring step. The interfacial tension is controlled by the parameter $A_{k}$, which is calibrated according to the desired macroscopic surface tension via the Laplace law.

### 2.3. Boundary condition

In this study, the boundary conditions are implemented to strictly match the physical setup of an infinite shear flow under an electric field.

#### 2.3.1. Flow Field Boundaries.

For the top and bottom boundaries, the Zou-He non-equilibrium bounce-back boundary condition is employed to simulate moving walls with constant velocities $u_{\omega }$ and $-u_{\omega }$. Taking the top boundary ($y=L_{y}$, velocity $u_{x}=u_{\omega }$, $u_{y}=0$) as an example, the unknown distribution functions $f_{\text{4}},f_{\text{7}},f_{\text{8}}$ are calculated as:


$$\begin{array}{c} f_{\text{4}}=f_{\text{2}} \end{array}$$ (27)

$$\begin{array}{c} f_{\text{7}}=f_{\text{5}}+\frac{\text{1}}{\text{2}}(f_{\text{1}}-f_{\text{3}})-\frac{\text{1}}{\text{2}}\rho u_{\omega } \end{array}$$ (28)

$$\begin{array}{c} f_{\text{8}}=f_{\text{6}}-\frac{\text{1}}{\text{2}}(f_{\text{1}}-f_{\text{3}})+\frac{\text{1}}{\text{2}}\rho u_{\omega } \end{array}$$ (29)

Where ρ is the local density at the wall. Similarly, the bottom boundary is set to $-u_{\omega }$ to generate a uniform shear rate $\gamma =2u_{\omega }/L_{y}$.

For the left and right boundaries, periodic boundary conditions are applied to simulate an infinite fluid domain in the flow direction. This is implemented by mapping the distribution functions as $f_{i}\left(0,y\right)=f_{i}\left(L_{x},y\right)$.

#### 2.3.2. Electric Field Boundaries.

The electric potential φ is solved using:

Left and right boundaries: Dirichlet boundary conditions, where $\varphi \left(0,y\right)=V_{a}$ and $\varphi \left(L_{x},y\right)=V_{b}$, acting as external electrodes.

Top and bottom boundaries: Neumann boundary conditions, where ∂φ/∂y=0, representing electrical insulation.

### 2.4. Pulse voltage formula

For unidirectional triangular pulse voltage, there is:


$$\begin{array}{c} U_{\text{1}}=\left\{ \begin{array}{c} @l@kt,\text{\textbackslash{}hspace\{1em}\;\;\;\;\}0<t<T/2\\ -kt+2U_{max},T/2<t<T \end{array}\right. \end{array}$$ (30)

Where $U_{\text{1}}$ is the voltage value of the unidirectional triangular pulsed electric field at time t, $U_{max}$ is the maximum voltage of the electric field, and T is a repetitive period. k is the slope, which is related to the maximum voltage $U_{max}$ and the period T, and is given by $k=2U_{max}/T$. And $U_{\text{1}}$ is a periodic unidirectional triangular pulse voltage with a period of T.

In this paper, a bidirectional triangular pulsed electric field is used. Based on Equation (31), we have:


$$\begin{array}{c} U_{\text{2}}=\left\{ \begin{array}{c} @c@U_{\text{1}},\text{\textbackslash{}hspace\{1em}\;\;\}0<t<T_{\text{0}}/2\\ -U_{\text{1}},\text{\textbackslash{}hspace\{1em}\;\}T_{\text{0}}/2<t<T_{\text{0}} \end{array}\right. \end{array}$$ (31)

Where $U_{\text{2}}$ is the voltage value of the bidirectional triangular pulse at time t. And $T_{\text{0}}$ is the total period, it contains a T periodic forward triangular pulse and a T periodic backward triangular pulse.

### 2.5. Model diagram

**Fig 1:**
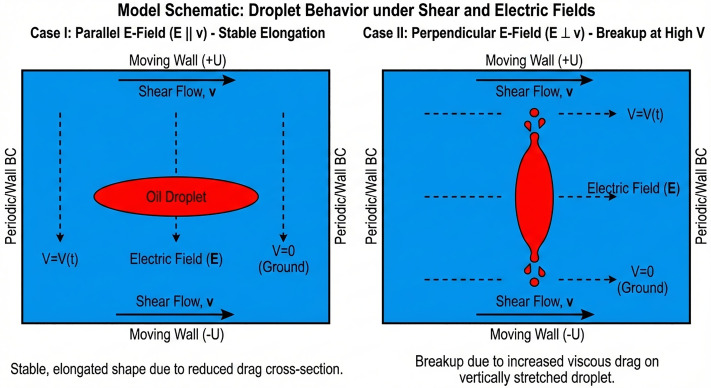
Schematic of the physical model for oil droplet behavior under the combined influence of shear and electric fields. (a) Case I: Parallel electric field resulting in stable elongation. (b) Case II: Perpendicular electric field leading to droplet breakup. The scalar boundary conditions for the electric potential (V=V(t) andV=0) and the velocity boundaries (+U and −U) are indicated.

As illustrated in Fig 1, a computational domain with periodic/wall boundary conditions is employed. The shear flow is generated by two parallel moving walls with velocities +U and−U, respectively. For the electric field, a scalar potential V(t) is applied at the top (or left/right) boundary, while the opposite boundary is grounded (V=0), creating a uniform electric field E.

## 3. Simulation results and discussion

This paper employs a computational grid of 256×256 units to simulate an oil-in-water (O/W) emulsion, with the electric field direction set parallel to the flow field, and the effects of gravity are neglected. At the same time, the momentum of the flow field is uniform and 0 in the initial state. At the initial conditions of the simulation setup, 9100 oil droplets with a diameter of 1 grid unit are randomly generated on the flow field grid. The total area occupied by these oil droplets accounts for approximately 10% of the entire flow field area. Consequently, the simulated emulsion concentration is approximately 10%. For the dimensionlessization of length, time, and voltage, there are


$$\begin{array}{c} \left\{ \begin{array}{c} @c@s=\frac{s^{\text{1}}}{10^{-6}m}\\ t=\frac{t^{\text{1}}}{10^{-4}s}\\ V=\frac{U}{10W/A} \end{array}\right. \end{array}$$ (32)

Where $s^{\text{1}}$, $t^{\text{1}}$ and U are the actual length, time and voltage, respectively. s, t, V are the dimensionless length, time and voltage.

Thus, the dimensionless velocity of the shear plate is:


$$\begin{array}{c} \gamma =\frac{A^{\text{1}}}{10^{\text{2}}} \end{array}$$ (33)

Where $A^{\text{1}}$ is the actual movement rate of the shear plate. The dielectric constant, conductivity and viscosity are dimensionless by the following methods.

${\varepsilon_{r}}^{\text{1}}$ and ${\varepsilon_{b}}^{\text{1}}$ represent the actual dielectric constants, whereas $\varepsilon_{r}$ and $\varepsilon_{b}$ are the nondimensionalized dielectric constants for the oil droplets and water, respectively. Thus,


$$\begin{array}{c} \varepsilon_{r}=\sqrt{\frac{{\varepsilon_{r}}^{\text{1}}}{{\varepsilon_{r}}^{\text{1}}+{\varepsilon_{b}}^{\text{1}}}},\text{\textbackslash{}hspace\{0.17em}\;\}\varepsilon_{b}=\sqrt{\frac{{\varepsilon_{b}}^{\text{1}}}{{\varepsilon_{r}}^{\text{1}}+{\varepsilon_{b}}^{\text{1}}}} \end{array}$$ (34)

${\sigma_{r}}^{\text{1}}$ and ${\sigma_{b}}^{\text{1}}$ denote the actual electrical conductivities of oil and water, respectively, whereas $\sigma_{r}$ and $\sigma_{b}$ represent the nondimensionalized electrical conductivities of the oil and water, respectively. Thus,


$$\begin{array}{c} \sigma_{r}=\sqrt{\frac{{\sigma_{r}}^{\text{1}}}{{\sigma_{r}}^{\text{1}}+{\sigma_{b}}^{\text{1}}}}\text{\textbackslash{}hspace\{0.17em}\},\text{\textbackslash{}hspace\{0.17em}\}\sigma_{b}=\sqrt{\frac{{\sigma_{b}}^{\text{1}}}{{\sigma_{r}}^{\text{1}}+{\sigma_{b}}^{\text{1}}}} \end{array}$$ (35)

${\eta_{r}}^{\text{1}}$ and ${\eta_{b}}^{\text{1}}$ are the actual viscosities of the oil droplets and water, respectively, whereas $\eta_{r}$ and $\eta_{b}$ represent the nondimensionalized viscosities of the oil and water, respectively. The relationship can be expressed as follows:


$$\begin{array}{c} \eta_{r}=\sqrt{\frac{{\eta_{r}}^{\text{1}}}{{\eta_{r}}^{\text{1}}+{\eta_{b}}^{\text{1}}}},\text{\textbackslash{}hspace\{0.17em}\;\}\eta_{b}=\sqrt{\frac{{\eta_{b}}^{\text{1}}}{{\eta_{r}}^{\text{1}}+{\eta_{b}}^{\text{1}}}} \end{array}$$ (36)

In this study, the parameters for the oil droplets are set to typical values for crude oil, with the actual parameters configured as Table 2 shows.

**Table 2:** The actual parameters of oil and water.

Actual parameters	Density	Absolute Permittivity	Electrical conductivity	Viscosity
Oil	$0.9\times 10^{\text{3}}kg/m^{\text{3}}$	$0.3\times 10^{-9}F/m$	$2\times 10^{-6}S/m$	0.0042Pa·s
Water	$1\times 10^{\text{3}}kg/m^{\text{3}}$	$7.8\times 10^{-9}F/m$	$5.5\times 10^{-6}S/m$	0.001Pa·s

And the nondimensionalized parameters configured as Table 3 shows.

**Table 3:** The Nondimensionalized parameters of oil and water.

Nondimensionalized parameters	Density	Absolute Permittivity	Electrical conductivity	Viscosity
Oil	0.9	0.19	0.52	0.87
Water	1.0	0.98	0.87	0.47

### 3.1. Physical interpretation of dimensionless parameters

To facilitate the comparison with experimental studies and industrial applications, we mapped the dimensionless Lattice Boltzmann units (lu, ts) to physical quantities through dimensional analysis. We assumed typical physical properties for a water-oil system: density $\rho \approx 1000{\text{kg/m}}^{\text{3}}$, kinematic viscosity $\nu \approx 10^{-6}{\text{m}}^{\text{2}}/\text{s}$, and interfacial tensionσ≈30mN/m.

As shown in Table 4, the selected shear rate corresponds to approximately 280.0 s^-1^ , and the electric field strength corresponds to approximately 3.0 kV/cm after dimensional conversion. It should be noted that these values are not uniquely defined due to the scaling freedom inherent in the lattice Boltzmann framework. Instead, they are chosen to fall within typical industrial ranges, ensuring the physical relevance of the simulation.

**Table 4:** Mapping between dimensionless simulation parameters and corresponding physical magnitudes (order-of-magnitude estimation based on characteristic scaling).

Quantity	Dimensionless Unit (LBM)	Conversion Factor (approx.)	Physical Value (Order of Magnitude)
Length Scale	1 lu (lattice unit)	$C_{l}\approx 2.7\mu m$	ChannelHeightH≈0.7mm
Time Scale	1 ts (time step)	$C_{t}\approx 3.6\mu s$	PulsePeriod(T=100)≈0.36ms
Shear Rate	$\gamma =0.001{\text{ts}}^{-1}$	$\overset{-1}{\underset{t}{C}}\approx 2.8\times 10^{\text{5}}{\text{s}}^{-1}$	$\gamma_{p}\approx 280{\text{s}}^{-1}$
Electric Field	$V=300(Ca_{E}\approx 0.7)$	$Derived\;from\;Ca_{E}$	E≈3kV/cm

The simulation in this paper has certain limitations. In reality, gravity and temperature are usually factors to be considered, which are ignored in this paper. At the same time, the parameters of water and oil in reality are not limited to a certain value. In addition, the model in this paper cannot deal with the case of turbulence. These factors lead to a certain difference between the simulation and the reality of this paper.

### 3.2. The evolution of emulsions in a shear flow field

**Fig 2:**
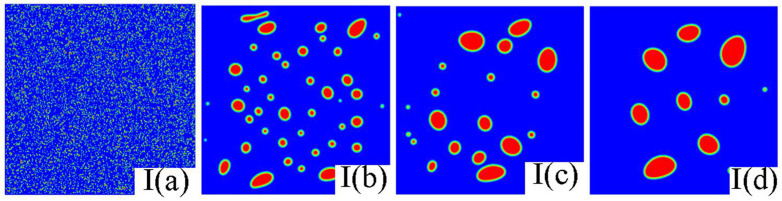
The evolution of sheared emulsion without electric field conditions. I(a)t=0, $I\left(b\right)t=3\times 10^{\text{4}}$, $I\left(c\right)t=7\times 10^{\text{4}}$, $I\left(d\right)t=2.4\times 10^{\text{5}}$.

In the settings of this article, the upper plate is set to move from left to right, while the lower plate moves from right to left, as shown in Fig 2. In this paper, the thickness of the plates is neglected. In Fig. 2. the shear rate γ is set to 0.001 (dimensionless), corresponding to approximately 280 s^-1^ in physical units.

In the shear flow field, the oil droplets and water are viscous. Therefore, once the upper and lower plates start moving, the oil droplets begin to move under the influence of shear forces and continuously collide with each other, as shown in Figure I(b). After the collision, the oil droplets will gather together stably due to the effect of surface tension. Moreover, oil droplets close to the upper and lower plates experience a stronger shear force. The aggregation speed of oil droplets in this area is faster than that of the oil droplets in the central region of the flow field. At this time, the aggregation behavior of the oil droplets is jointly affected by shear forces and surface tension. When the oil droplets coalesce into larger droplets, they move clockwise around the center of the flow field under the influence of shear forces, as shown in Figure I(d). At this point, the shear force counteracts the surface tension, with the shear effect being stronger than the surface tension. The evolution of the emulsion reaches a stable state, and the oil droplets no longer aggregate.

### 3.3. The evolution of emulsions in a pulsed electric field coupled with a shear flow field

In this section, the upper plate is set to move from left to right, while the lower plate moves from right to left. Additionally, this paper employs a bidirectional triangular pulsed electric field.

#### 3.3.1. The evolution of emulsions under different shear rates.

**Fig 3:**
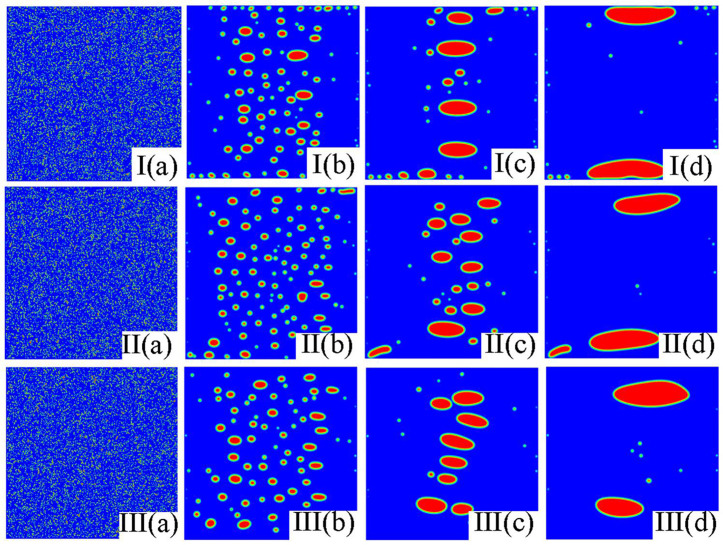
Evolution of emulsions under different shear rates, where V = 300. I(a)t=0, $I\left(b\right)t=4\times 10^{\text{4}}$, $I\left(c\right)t=1\times 10^{\text{5}}$, $I\left(d\right)t=2.6\times 10^{\text{5}}$, for shear rate is $\gamma =1\times 10^{-5}$, II(a)t=0, $II\left(b\right)t=2\times 10^{\text{4}}$, $II\left(c\right)t=7\times 10^{\text{4}}$, $II\left(d\right)t=1.7\times 10^{\text{5}}$, for $\gamma =1\times 10^{-4}$, III(a)t=0, $III\left(b\right)t=2\times 10^{\text{4}}$, $III\left(c\right)t=5.3\times 10^{\text{4}}$, $III\left(d\right)t=1\times 10^{\text{5}}$, for $\gamma =5\times 10^{-4}$.

Fig 3 illustrates the evolution of emulsions under different shear rates. The upper and lower plates have the same shear rate but in opposite directions, and the upper plate moves from left to right. The shear rates $\gamma =1\times 10^{-5}$, $\gamma =1\times 10^{-4}$, and $\gamma =5\times 10^{-4}$. Bidirectional triangular pulsed electric fields are applied on the left and right sides of the flow field, with the initial direction of the electric field from left to right. The dimensionless voltage V = 300.

The emulsion evolution diagram shows: a) When the shear rate $\gamma =1\times 10^{-5}$, the electric field force is greater than the shear force. Therefore, oil droplets tend to aggregate in a direction parallel to the electric field. Eventually, two large oil droplets form at the upper and lower boundaries of the flow field. b) When the shear rate increases to $\gamma =1\times 10^{-4}$ and $\gamma =5\times 10^{-4}$, the effect of shear force causes the long axis of the oil droplets to form an angle with the direction of the electric field. At the same time, the distribution of oil droplets during the intermediate evolution process becomes more dense. c) When the shear rate increases, the shear force drives oil droplets to aggregate more towards the center of the flow field. However, during the evolution process, the electric field force always plays a dominant role. As a result, oil droplets eventually aggregate to form two large oil droplets in the upper and lower parts of the flow field. Additionally, a higher shear rate causes the two large oil droplets to move closer to the center of the flow field.

The results show: a) When the effect of shear force is much smaller than that of the electric field force, the aggregation of oil droplets mainly occurs in the direction parallel to the electric field. b) An increase in shear force promotes more aggregation of oil droplets in the direction perpendicular to the electric field. c) In the coupled shear emulsion with bidirectional pulsed electric fields, increasing the shear force can cause oil droplets to move towards the center of the flow field. This indicates that increasing the shear force can enhance the demulsification effect of the coupled fields on the emulsion.

#### 3.3.2. The direction of the electric field is perpendicular to the direction of shear.

**Fig 4:**
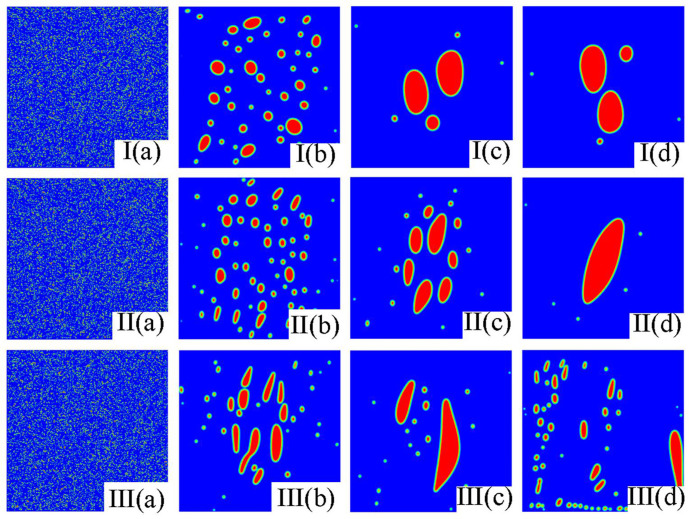
Evolution of sheared emulsion under different intensities of bidirectional triangular pulsed electric fields, where the electric field direction is perpendicular to the shear direction. I(a)t=0, $I\left(b\right)t=3\times 10^{\text{4}}$, $I\left(c\right)t=2.51\times 10^{\text{5}}$, $I\left(d\right)t=2.63\times 10^{\text{5}}$, for V = 100, II(a)t=0, $II\left(b\right)t=1.5\times 10^{\text{4}}$, $II\left(c\right)t=4.8\times 10^{\text{4}}$, $II\left(d\right)t=1.02\times 10^{\text{5}}$, for V = 300, III(a)t=0, $III\left(b\right)t=2.1\times 10^{\text{4}}$, $III\left(c\right)t=4\times 10^{\text{4}}$, $III\left(d\right)t=9.25\times 10^{\text{4}}$, for V = 400.

In Fig 4, the dimensionless voltage values are set to 100, 300, and 400. The direction of the electric field is from top to bottom, perpendicular to the direction of shear. And the shear rate γ is set to 0.001. Due to the polarization effect of the pulsed electric field on oil droplets, charges will accumulate at the ends of the oil droplets. Therefore, the two ends of the charged oil droplets will be affected by the electric field force. This electric field force will prompt the oil droplets to move or deform along the direction of the electric field. Meanwhile, the shear force will also induce movement or deformation of the oil droplets. The evolution of emulsion under different electric field intensities shows the following: a) At V = 100, the oil droplets tend to gather towards the middle of the flow field under the influence of electric field force and shear force. Eventually, they orbit around the center of the flow field under the action of shear force and reach a stable state. After that, the oil droplets no longer aggregate. b) At V = 300, the increased electric field intensity accelerates the aggregation of oil droplets, eventually leading to the formation of a stable large oil droplet in the middle of the flow field. Under the influence of shear force and electric field force, the oil droplets take on the shape of an inclined elliptical cone. c) At V = 400, the even greater electric field intensity causes the oil droplets to deform more significantly in the direction of the electric field. However, after the oil droplets have aggregated sufficiently, they undergo fragmentation. This phenomenon leads to the reformation of the aggregated oil droplets into a greater number of smaller oil droplets. In addition, this phenomenon is characterized by periodic aggregation and breakage of oil droplets.

The results show the following: a) The combined action of electric field force and shear force accelerates the aggregation speed of oil droplets. b) When the effect of shear force is greater than that of the electric field force, the oil droplets tend to move stably around the center of the flow field. This hinders further aggregation of the oil droplets. c) A greater electric field intensity disrupts the final stable state of the oil droplets, encouraging them to continue aggregating. However, an excessively high electric field intensity can cause significant deformation of the oil droplets. At this point, the combined effect of the electric field force and shear force exceeds the surface tension of the oil droplets. As a result, the oil droplets undergo fragmentation.

#### 3.3.3. The direction of the electric field is parallel to the direction of shear.

**Fig 5:**
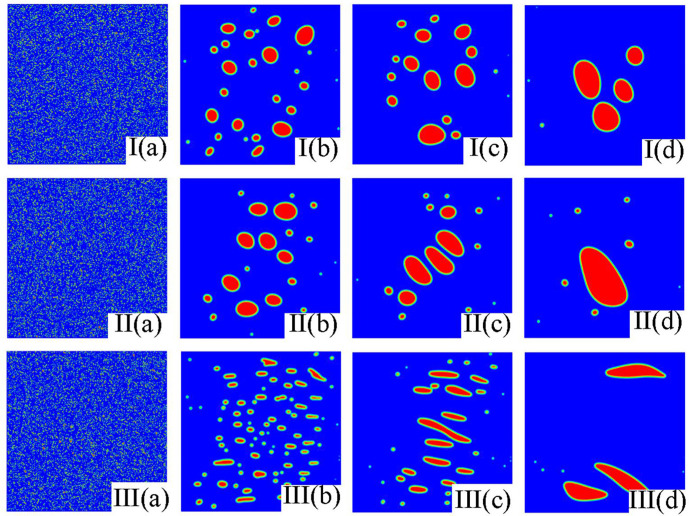
Evolution of sheared emulsion under different intensities of bidirectional triangular pulsed electric fields, where the electric field direction is parallel to the shear direction. I(a)t=0, $I\left(b\right)t=4.4\times 10^{\text{4}}$, $I\left(c\right)t=9\times 10^{\text{4}}$, $I\left(d\right)t=1.74\times 10^{\text{5}}$, for V = 100, II(a)t=0, $II\left(b\right)t=5\times 10^{\text{4}}$, $II\left(c\right)t=6.5\times 10^{\text{4}}$, $II\left(d\right)t=9\times 10^{\text{4}}$, for V = 200, III(a)t=0, $III\left(b\right)t=1\times 10^{\text{4}}$, $III\left(c\right)t=2\times 10^{\text{4}}$, $III\left(d\right)t=5\times 10^{\text{4}}$, for V = 500.

In Fig 5, the dimensionless voltage values are set to 100, 200, and 500. The direction of the electric field is from left to right, parallel to the direction of shear. And the shear rate γ is set to 0.001.

When the electric field direction is parallel to the shear direction, the evolution of the emulsion shows different results: a) At V = 100, the electric field force and shear force promote the aggregation of oil droplets. The aggregation trend of the oil droplets tends towards the center of the flow field. Since the effect of the electric field force is less than the shear force and the surface tension of the oil droplets, the oil droplets eventually move stably around the center of the flow field and cannot continue to aggregate. b) At V = 200, the aggregation trend of the oil droplets is the same. However, the increase in the electric field force disrupts the previous stable state. Eventually, a stable large oil droplet forms in the lower part of the flow field. c) At V = 500, the effect of the electric field force is much greater than the surface tension of the oil droplets. Therefore, the oil droplets undergo significant deformation, taking on an elongated shape. At the same time, because the electric field force is greater than the shear force, the trend of oil droplets moving in the direction of the electric field is stronger. However, this phenomenon hinders the aggregation of oil droplets perpendicular to the direction of the electric field. Eventually, oil droplets aggregate at the upper and lower parts of the flow field, forming elongated large oil droplets.

The results indicate the following: a) When the electric field force is less than the shear force and the surface tension of the oil droplets, the oil droplets tend to form a stable state that moves around the center. b) An increase in the electric field force can promote more complete aggregation of the oil droplets. c) When the electric field direction is parallel to the shear direction, the effect of the electric field force causing deformation of the oil droplets counteracts the shear force. The combined effect of both is less than the surface tension of the oil droplets. Therefore, an excessive electric field force does not lead to the rupture of the oil droplets. Compared to when the electric field direction is perpendicular to the shear direction, this suggests that a greater electric field intensity can be used for demulsification when the electric field direction is parallel to the shear direction.

## 4. Analysis of the evolution of streamlines

### 4.1. Shear field influence on the evolution of streamlines in a flow field

**Fig 6:**
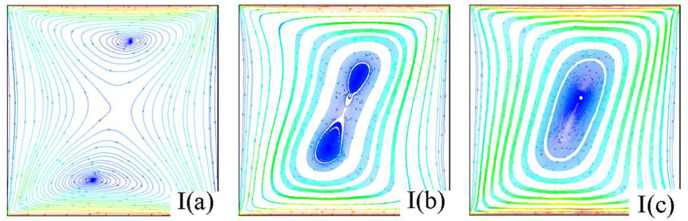
Flow streamlines of sheared emulsion without an electric field. And the shear rate γ is set to 0.001. $I\left(a\right)t=1\times 10^{\text{3}}$, $I\left(b\right)t=5\times 10^{\text{3}}$, $I\left(c\right)t=1.1\times 10^{\text{4}}$.

Fig 5 displays the changes in the streamlines of the flow field. In this scenario, no electric field is applied at the sides of the flow field, and the upper plate moves at a constant speed from left to right, while the bottom plate moves in the opposite direction. Where the shear rate γ is set to 0.001. Under the influence of shear force, two identical vortices appear at the top and bottom of the flow field, as shown in I(a). Subsequently, these two vortices, driven by shear force, continuously move closer to each other. Eventually, they merge into a single stable vortex in the middle of the flow field. The results indicate that the shear field tends to drive the flow field towards a stable state, with oil droplets in the flow field moving stably around the center after insufficient aggregation. This outcome prevents further aggregation of the oil droplets (Fig 6).

### 4.2. Evolution of streamlines in a flow field under the action of coupled bidirectional triangular pulsed electric and shear fields

**Fig 7:**
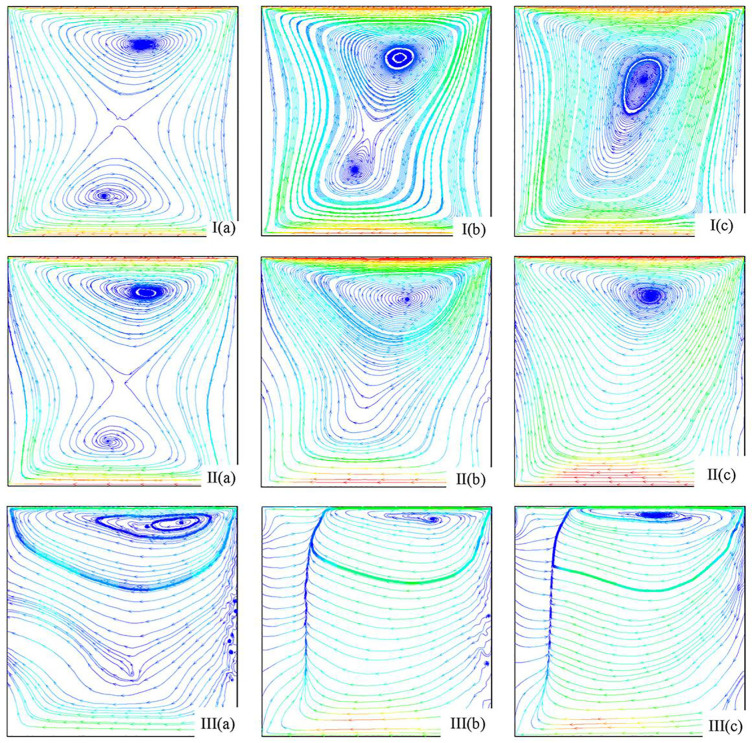
Evolution of flow streamlines in the flow field under the coupled action of bidirectional triangular pulsed electric fields and shear fields, with the electric field direction parallel to the shear direction. Where the shear rate γ is set to 0.001. $I\left(a\right)t=1\times 10^{\text{3}}$, $I\left(b\right)t=3\times 10^{\text{3}}$, $I\left(c\right)t=1\times 10^{\text{4}}$, $II\left(a\right)t=1\times 10^{\text{3}}$, $II\left(b\right)t=2\times 10^{\text{3}}$, $II\left(c\right)t=6\times 10^{\text{4}}$, $III\left(a\right)t=1\times 10^{\text{3}}$, $III\left(b\right)t=2\times 10^{\text{3}}$, $III\left(c\right)t=8\times 10^{\text{3}}$.

This article sets up bidirectional triangular pulsed electric fields on the left and right sides of the flow field, with the initial direction of the electric field being from left to right. Both the upper and lower plates move at a constant speed in opposite directions. Where the upper plate moving from left to right and the shear rate γ is set to 0.001. Fig 7 shows the evolution of streamlines in the flow field under these conditions.

a) As shown in Fig.6 I(a), I(b), and I(c), the pulsed electric field at this time is V = 100. Due to the initial direction of the pulsed electric field being from left to right, this phenomenon leads to an acceleration of the flow in the same direction as the electric field and a deceleration of the flow in the opposite direction. At the same time, the densely packed streamlines in the upper part of the flow field are in the same direction as the electric field, while the densely packed streamlines in the lower part are in the opposite direction. Therefore, the pulsed electric field causes the flow field to have two uneven vortices, with the streamlines in the upper part of the flow field being denser than those in the lower part, as shown in I(a). At this time, due to the electric field force being less than the shear force, the two vortices in the flow field eventually merge into one, and a stable state is ultimately formed in the area close to the upper part of the flow field. However, this stable state hinders further aggregation of oil droplets.
b) When V = 200, the electric field force has a stronger effect on accelerating and decelerating fluid motion. As a result, the two vortices initially generated in the flow field are more unevenly distributed, as shown in II(a). Under the action of shear force, the two vortices merge. The position of the merged vortex is closer to the upper plate, as shown in II(c). At this time, the effect of the electric field force is stronger than that of the shear force, so the flow field distribution around the vortex is not in a stable state. This phenomenon promotes the aggregation of oil droplets from different parts of the flow field.
c) When V = 500, the effect of the electric field force is far stronger than that of the shear force. Consequently, the formation of vortices in the upper part of the flow field accelerates, as shown in III(a). Due to the effects of viscosity and inertia, the change in the internal flow direction lags behind the change in the electric field direction. Therefore, when the electric field direction changes, the direction of the electric field force becomes antagonistic to the flow direction of the fluid. The higher the electric field intensity, the more pronounced this antagonism becomes. As shown in III(b) and III(c), the streamline direction in the left part of the flow field is opposite to that in the right part. After converging, the flow direction turns towards the upper part of the flow field, but the fluid motion slows down afterward. This phenomenon hinders the mutual aggregation of oil droplets in the upper and lower halves of the flow field.

The results indicate that when the electric field direction is parallel to the shear direction, the bidirectional triangular pulsed electric field promotes the formation of an uneven distribution in the flow field by accelerating or decelerating the flow in different parts of the field. This increases the probability of collisions between oil droplets from different parts of the flow field, leading to more complete aggregation of the oil droplets. However, an excessively high electric field intensity can result in the presence of fluids with different flow directions in the flow field and the formation of areas with slow flow velocities. This phenomenon hinders the mutual aggregation of oil droplets.

## 5. Quantitative analysis of the demulsification effect of pulsed electric fields on emulsions

Under the action of electric field, the oil droplets appear electrophoretic aggregation and shock aggregation, and the number of oil droplets with a large area gradually increases. At the same time, the shape of oil droplets changes periodically along the direction of electric field. Due to the large number of oil droplets in the emulsion, counting the area change and morphological change of each oil droplet requires more computing resources. Therefore, this paper uses the method of counting the ratio of area to perimeter of all oil droplets to study and analyze the degree of aggregation and morphological change in the process of oil droplet aggregation. There are:


$$\begin{array}{c} Dr=\frac{S_{r}}{L_{r}} \end{array}$$ (37)

where Dr is the ratio of the total area to the total perimeter of the oil droplets, $S_{r}$ is the total area of the oil droplets, and $L_{r}$ is the total perimeter of the oil droplets. Assume that $R_{ave}$ is the average radius of oil droplets, there is:


$$\begin{array}{c} S_{r}=\pi \overset{\text{2}}{\underset{ave}{R}} \end{array}$$ (38)

$$\begin{array}{c} L_{r}=2\pi R_{ave} \end{array}$$ (39)

Substituting the above two formulas into (47), we can obtain:


$$\begin{array}{c} Dr=\frac{R_{ave}}{\text{2}} \end{array}$$ (40)

It can be seen that Dr is proportional to $R_{ave}$ under the assumption of ideal spherical droplets. In the highly dynamic coupled electric-shear field, droplets experience both coalescence (which increases Dr) and electro-deformation or shear stretching (which increases the perimeter and decreases Dr). Therefore, without applying a shape correction factor, the change trend of Dr acts as a composite morpho-dynamic index. It not only reflects the aggregation degree of oil droplets but also actively penalizes excessive, unstable morphological deformation, providing a comprehensive metric for the ultimate stability of the emulsion. During the aggregation process of two oil droplets, the total area of the two oil droplets remains unchanged, while the total perimeter increases, which leads to Dr becoming larger. When Dr is on the rise as a whole, it shows that the aggregation behavior of oil droplets is dominant at this time. When the aggregation behavior of oil droplets is completed, the shape of oil droplets changes under the action of electric field force. This effect causes the perimeter of the oil droplets to change, while the area of the oil droplets remains unchanged. At this time, the change of Dr shows the degree of shape change of oil droplets. The smaller the Dr, the greater the degree of change in the shape of the oil droplets. Therefore, the change of Dr can quantitatively analyze the aggregation behavior and shape change of oil droplets at different stages.

### 5.1. Quantitative analysis of emulsion evolution under no electric field and bidirectional triangular pulsed electric field control

**Fig 8:**
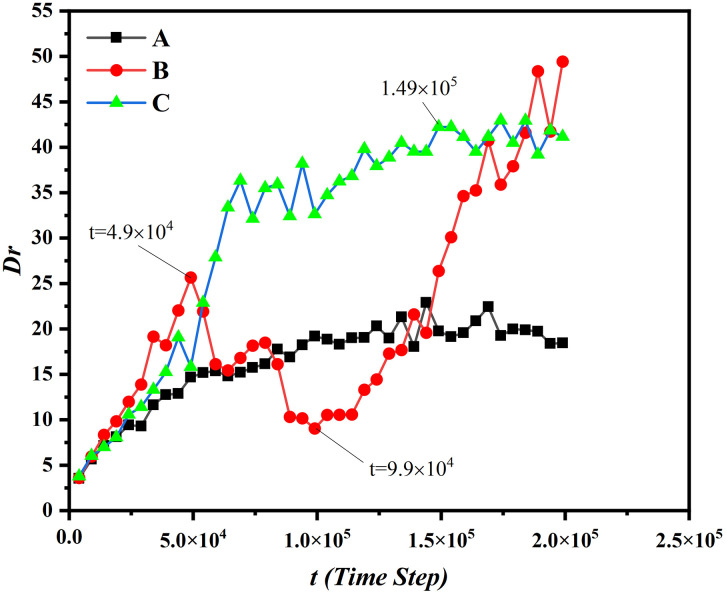
Changes in the Dr value of emulsion in shear flow without an electric field and in bidirectional triangular pulsed electric fields coupled with shear fields in different directions. And the shear rate γ is both set to 0.001. Fig 8(A) represents shear flow without an electric field, Fig 8(B) represents the bidirectional triangular pulsed electric field coupled with the shear field, where the electric field direction is perpendicular to the shear direction, V = 400. Fig 8(C) represents the bidirectional triangular pulsed electric field coupled with the shear field, where the electric field direction is parallel to the shear direction, V = 300.

As shown in Fig 8, the quantitative results indicate:

a) As Fig 8A shows, when no pulsed electric field is applied in the shear flow field, the curve of Dr grows slowly and the overall increase is less than the other two curves. This suggests that the aggregation efficiency of oil droplets in emulsion under shear without an electric field is lower than that in emulsion under bidirectional triangular pulsed electric field control.
b) At $t\leq 4.9\times 10^{\text{4}}$, the growth rate of curve Fig 8(C) is less than that of Fig 8(B). This indicates that, during this period, the demulsification efficiency of the emulsion with the electric field direction perpendicular to the shear direction is higher than that with the electric field direction parallel to the shear direction. However, due to the breakage of oil droplets under the action of electric field force and shear force, the Fig 8(B) curve rapidly decreases afterward. At the same time, the Fig 8(C) curve rises quickly, reaching an inflection point at $t=1.49\times 10^{\text{5}}$, indicating that the oil droplets in the emulsion have completed aggregation.
c) The Fig 8(B) curve rapidly decreases after the oil droplets break, reaching the lowest point at $t=9.9\times 10^{\text{4}}$, after which the Dr value begins to rise gradually. This indicates that the oil droplets in the emulsion begin to re-aggregate. However, overall, the Fig 8(C) curve reaches a stable state more quickly and grows at a faster rate. Therefore, when the direction of the bidirectional triangular pulsed electric field is parallel to the shear direction, the demulsification efficiency of the emulsion is more stable and efficient.

### 5.2. Quantitative analysis of emulsion evolution under different shear rates

**Fig 9:**
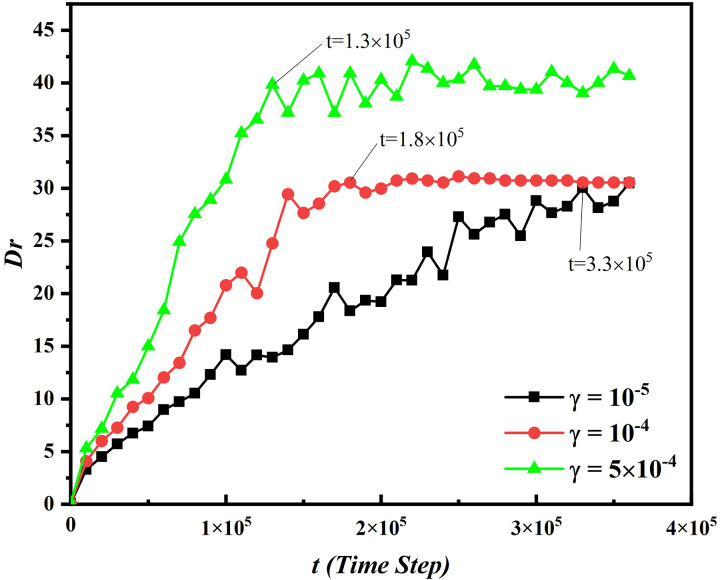
Variation of Dr values under different shear rates, where the voltage of the bidirectional triangular pulsed electric field V = 300.

Fig 9 illustrates the variation of Dr values for emulsions under different shear fields coupled with bidirectional triangular pulsed electric fields. The upper and lower plates have shear rates of the same magnitude but in opposite directions. Additionally, the dimensionless voltage of the bidirectional triangular pulsed electric field is V = 300, with the initial direction of the electric field from left to right.

The quantitative results show:

a) At a shear rate of $\gamma =5\times 10^{-4}$, the curve reaches an inflection point at $t=1.3\times 10^{\text{5}}$. At a shear rate of $\gamma =10^{-4}$, the curve reaches an inflection point at $t=1.8\times 10^{\text{5}}$. At a shear rate of $\gamma =10^{-5}$, the Dr value of the curve reaches an inflection point at $t=3.3\times 10^{\text{5}}$. This indicates that the overall morpho-dynamic evolution (coalescence and stable deformation) of oil droplets in the emulsion is the most significant at $\gamma =5\times 10^{-4}$. Therefore, increasing the shear rate can improve the aggregation speed of oil droplets.
b) The curve for $\gamma =5\times 10^{-4}$ has a higher Dr value throughout the entire evolution stage compared to the other two curves. This indicates that the aggregation degree of oil droplets in the emulsion is the highest at $\gamma =5\times 10^{-4}$. Therefore, increasing the shear rate can enhance the aggregation degree of oil droplets.
c) The curve for $\gamma =10^{-4}$ approaches a straight line after reaching the inflection point, and the rate of change of Dr tends to 0. This indicates that the shape of the oil droplets has reached a stable state and no longer changes due to the effects of electric and shear forces. At this point, the effects of shear force and electric field force on the oil droplets are in balance.

### 5.3. Quantitative analysis of the evolution of sheared emulsion under different intensities of bidirectional triangular pulsed electric fields

**Fig 10:**
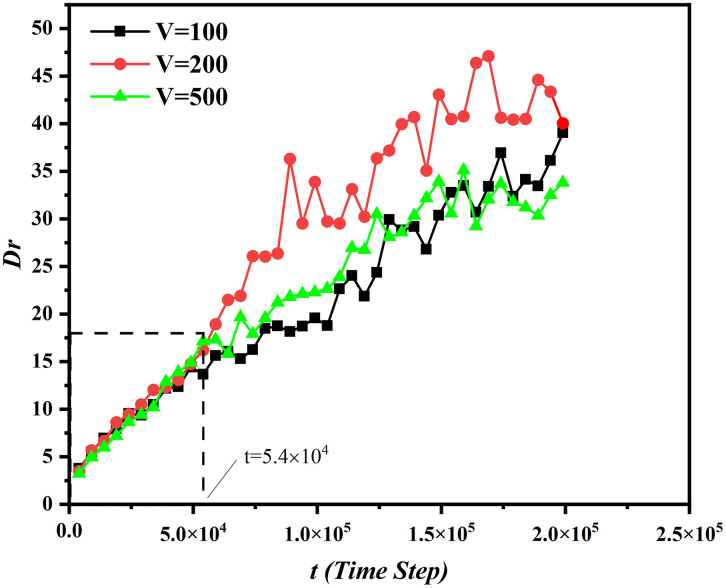
Changes in the Dr value of sheared emulsion under different intensities of bidirectional triangular pulsed electric fields, with the electric field direction parallel to the shear direction. And the shear rate γ is both set to 0.001.

Fig 10 shows the changes in the Dr value of sheared emulsion under different intensities of bidirectional triangular pulsed electric fields. And the shear rate γ is both set to 0.001. The quantitative results show:

a) At the initial time, the change in all three curves is the same. At this point, the aggregation speed of oil droplets in the emulsion under the influence of the three electric field intensities is the same. The demulsification effect of the three electric field intensities on the emulsion is the same.
b) Afterwards, the curve for V = 200 accelerates in growth rate and remains higher than the other two curves thereafter. This indicates that at V = 200, the demulsification effect of the bidirectional triangular pulsed electric field on sheared emulsion is higher than that at V = 100 and V = 500.
c) The curves for V = 100 and V = 500 show similar trends throughout the entire period, indicating that the demulsification effects of the two electric field intensities on sheared emulsion are similar. This phenomenon demonstrates that when the electric field direction is parallel to the shear direction, high electric field intensity can actually hinder the demulsification efficiency of the bidirectional pulsed electric field on the emulsion.

## 6. Conclusion

In this paper, the evolution of emulsion in coupled bidirectional triangular pulsed electric field and shear field is numerically simulated. The parameters of water and crude oil are used to set the parameters, which can provide some reference for the separation of oil-containing dilute emulsion. At the same time, this paper reveals the mechanism of bidirectional pulsed electric field affecting the aggregation of oil droplets in shear emulsion by analyzing the change of streamline diagram of flow field. In addition, this paper quantitatively analyzes the demulsification effect of the bidirectional triangular pulsed electric field on the shear emulsion. The simulation results show that:

a) When the electric field is not applied in the shear flow field, the oil droplets tend to move stably around the center of the flow field under the action of shear force, which to some extent hinders the full aggregation of the oil droplets.
b) The bidirectional pulsed electric field alters the velocity distribution of the flow field, disrupting the ultimate stable motion state of the oil droplets. This encourages the oil droplets to further aggregate. Quantitative analysis shows that under the optimal conditions (V = 300 andγ=0.001), the composite morpho-dynamic index (Dr) reached 11.5. This represents a 4.7-fold increase compared to the pure shear field (Dr≈2.0) and a 109% improvement relative to the low-voltage condition (V = 100), demonstrating a superior balance between rapid droplet coalescence and stable morphological evolution.
c) When the electric field direction is perpendicular to the shear direction, high electric field intensity can cause the oil droplets to break after aggregation. However, when the electric field direction is parallel to the shear direction, the oil droplets exhibit better stability under high electric field intensity.
d) High-intensity bidirectional pulsed electric fields induce low-velocity areas in the flow field, which hinder the demulsification efficiency of the bidirectional pulsed electric field on sheared emulsions. Therefore, at a lower, more appropriate electric field intensity, the demulsification effect of the bidirectional pulsed electric field on sheared emulsions is better.
e) In addition the current model assumes a laminar regime, which is consistent with the micro-scale droplet interactions studied. For industrial applications involving high-Reynolds-number turbulence, the model would require integration with Large Eddy Simulation (LES) methods to account for stochastic turbulent fluctuations.

## Supporting information

S1 Data Raw data for quantitative analysis. The Excel file contains three worksheets: a) Worksheet “fig8”: Dr values corresponding to Fig. 8. Column A: Time step; Column B: Pure shear field (no electric field); Column C: Electric field perpendicular to shear direction, V=400; Column D: Electric field parallel to shear direction, V=300. b)Worksheet “fig9”: Dr values corresponding to Fig. 9. Column A: Time step; Column B: Shear rate = 1×10^-5^; Column C: Shear rate = 1×10^-4^; Column D: Shear rate = 5×10^-4^. All conditions with V=300. c) Worksheet “fig10”: Dr values corresponding to Fig. 10. Column A: Time step; Column B: V=100; Column C: V=200; Column D: V=500. Electric field direction parallel to shear direction, shear rate = 0.001.
(XLSX)
