# Supplementary material for: Simulation of emulsion evolution in shear flow field under the control of bidirectional pulsed electric field
Source: PLoS One. 2026 Jul 10;21(7):e0336345. doi: 10.1371/journal.pone.0336345 (PMC13354001; doi:10.1371/journal.pone.0336345)
Supplement: Attachment — Submitted filename: Reviewer comments.pdf [file pone.0336345.s002.pdf]

This manuscript establishes an LBM-based coupled electric–shear model to study droplet coalescence under a bidirectional triangular pulsed electric field. The topic is relevant to demulsification, and the manuscript is generally well-structured with clear qualitative trends.

1.(Section 2 “Numerical method”, electrostatics part) Poisson equation uses $\varepsilon_b$(water permittivity), and Eqs. (1)–(3) define $\nabla^2\varphi$, $E = -\nabla\varphi$, and Maxwell-stress-based force. Clarify why the present Poisson formulation is appropriate for an oil–water two-phase system and how permittivity contrast is handled。

2. (Section 3 “Simulation results and discussion”, initial setup): 256×256 grid, 9100 droplets, droplet diameter = 1 grid unit, ~10% area fraction. Please include at least one resolution or initial droplet-size sensitivity test (e.g., droplet diameter higher grid resolution) to demonstrate that the main conclusions (coalescence rate/Dr trends) are not dominated by lattice-scale effects.

3. Nondimensionalization needs clearer physical interpretation。Section 3, Eqs. (38)–(41) give nondimensionalization of length/time/voltage and material properties. It is difficult to infer what physical magnitudes (electric field strength, shear rate) correspond to the chosen nondimensional settings and whether they match typical demulsification conditions.Please add a short explanation (or a small table) mapping dimensional to nondimensional quantities and provide the approximate physical orders of magnitude for electric field strength and shear rate.

4. Breakup and “periodic aggregation–breakage” require an objective quantitative criterion.Section 3.2.2 reports fragmentation at higher field intensity and mentions periodic aggregation–breakage. The discussion states breakup occurs when electric + shear effects exceed surface tension. The current presentation is mainly qualitative; a reproducible breakup criterion and quantification of “periodicity” are missing.

5. Table 1 reports “Dielectric constant” in F/m (oil: $0.3\times10^{-9}$ F/m; water: $7.8\times10^{-9}$ F/m). F/m corresponds to absolute permittivity, while “dielectric constant” is often interpreted as relative permittivity (dimensionless).Please use consistent terminology (permittivity vs dielectric constant) and ensure units match the intended quantity.

6. Fig. 8–9 quantitative discussion compares shear rates and highlights an “optimal” trend. Streamline analysis explains the flow-direction change lags behind electric-field switching due to inertia/viscosity. The rationale for chosen shear-rate points and their relation to $T_0$/switching frequency is not discussed.
