# Supplementary material for: Simulation of emulsion evolution in shear flow field under the control of bidirectional pulsed electric field
Source: PLoS One. 2026 Jul 10;21(7):e0336345. doi: 10.1371/journal.pone.0336345 (PMC13354001; doi:10.1371/journal.pone.0336345)
Supplement: Attachment — Submitted filename: Response to reviewers.doc [file pone.0336345.s004.doc]

Dear Editors and Reviewers,

We appreciate the opportunity to revise our manuscript titled " Simulation of emulsion evolution in shear flow field under the control of bidirectional pulsed electric field" and are grateful for the insightful comments provided by the reviewers. We have carefully addressed all the comments of reviewers and revised the manuscript accordingly. Attached, you will find: (1) a detailed response letter outlining the changes made in response to the reviewers' suggestions, (2) a revised version of our manuscript with tracked changes, and (3) a clean version of our revised manuscript, free of any tracked changes.

As requested, we have meticulously addressed all reviewer comments and comprehensively improved the manuscript accordingly. Specifically: (1) The manuscript has been reformatted strictly following template; (2) The funding information has been updated. All revisions are marked in red in the manuscript; (3) The funding information has been updated in the cover letter to align with the acknowledgment section. Please let us know if any further modifications are needed.

sincerely yours,

Heping Wang

**Response to the reviewers' comments:**

Reviewer #1：

- Question Q1. What is the background for exploring the impact of the combined action of BPEF and shear field? It should be introduced briefly in the abstract.

- Question Q2. Was the O/W emulsion used as the research object?

- Question Q3. In abstract and conclusion, some quantitative results must be given.

- Question Q4. How did you distinguish the vectors and scalars?

- Question Q5. For some equations used in this work, some references are necessary.

- Question Q6. What is the value of interfacial tension for the oil and water?

- Question Q7. A diagram of model should be given, including the scalar, boundary conditions for the shear and electric fields.

**Response:** Thank you very much for your time in reviewing our manuscript. We sincerely appreciate your constructive suggestions, which have helped us significantly improve the quality of our work. We are also grateful for the opportunity to revise the manuscript based on your feedback. In this revision, we have carefully addressed your concerns point-by-point. We hope that the revised manuscript now meets your expectations and the journal’s standards.

**- Question Q1. What is the background for exploring the impact of the combined action of BPEF and shear field? It should be introduced briefly in the abstract.**

**Response:** We agree. In the revised Abstract, we have added the background information. We explained that while pure shear fields rely on random collisions (inefficient for fine droplets) and continuous electric fields can cause short-circuits, the synergy of BPEF and shear fields provides both mechanical dynamics and polarization forces to break the interfacial film resistance more effectively.

**Copy:** Electrostatic demulsification is a widely used technique for treating high-water-cut emulsions (O/W type). However, in high-water-cut emulsions, continuous electric fields often lead to short-circuiting and chain formation. Meanwhile, pure shear fields rely on random collisions, which is inefficient for fine droplets.

**- Question Q2. Was the O/W emulsion used as the research object?**

**Response:** Yes, the research object in this simulation is an O/W (Oil-in-Water) emulsion. We have explicitly stated this in the revised Abstract. The simulation generates oil droplets (dispersed phase) in a continuous water phase, with an oil concentration of approximately 10%.Revision: See Abstract (Line 2): "...treating high-water-cut emulsions (O/W type)." and Section 3 (Paragraph 1):This paper employs a computational grid of 256×256 units to simulate an oil-in-water (O/W) emulsion,

**- Question Q3.In abstract and conclusion, some quantitative results must be given.**

a) **Response:** We have included specific quantitative data in the revised manuscript to demonstrate the efficiency of the proposed method.In Abstract: "Under the optimal electric potential of V=300 and shear rate of$\gamma=0.001$, the average aggregation degree ($Dr$) reached 11.5, which represents a 4.7-fold increase compared to the pure shear field ($Dr\approx2.0$) and a 109% improvement compared to the low-voltage condition (V=100)."

In conclusion: “This represents a 4.7-fold increase compared to the pure shear field ($Dr\approx2.0$) and a 109% improvement relative to the low-voltage condition (V=100), demonstrating a superior balance between rapid droplet coalescence and stable morphological evolution.”

**- Question Q4.How did you distinguish the vectors and scalars?**

**Response:** We have added a "Symbol" table before the Introduction to clearly define the physical meanings and types of all variables.

| Symbol | Physical meaning | Type |
| --- | --- | --- |
| $\rho$ | Density | Scalar |
|  | Electric potential | Scalar |
| $\varepsilon$ | Permittivity | Scalar |
| $\sigma$ | Interfacial tension | Scalar |
| $\mu$ | Dynamic viscosity | Scalar |
| $\gamma$ | Shear rate | Scalar |
| $V$ | Dimensionless voltage | Scalar |
| $Dr$ | Aggregation degree | Scalar |
| $A$ | Droplet area | Scalar |
| $P$ | Droplet perimeter | Scalar |
| $t$ | Time | Scalar |
| $u$ | Velocity | Vector |
| $E$ | Electric field | Vector |
| $F$ | Total body force | Vector |
| $F_{e}$ | Electric field force | Vector |
| $F_{s}$ | Surface tension force | Vector |
|  | Gradient operator | Vector operator |

**- Question Q5.For some equations used in this work, some references are necessary.**

**Response:**We have added the necessary citations for the governing equations, including the Poisson equation for heterogeneous media, the Maxwell stress tensor, and the fundamental Lattice Boltzmann (LBM) evolution equations (e.g., [29] for the BGK model, [30] for the color model).

**Copy**: Due to the periodic change of the electric field, the electromagnetic induction effect will produce a magnetic field. However, in this paper, the current generated by the internal charge flow of the fluid is very small, so the influence of the magnetic field force is ignored. For the calculation of electric field distribution in the flow field, the central difference method is used in this paper. The electric potential distribution within the computational domain is governed by the Poisson equation for a heterogeneous dielectric medium ^[29]^

Therefore, the LBM control equation in the form of BGK can be obtained ^[30]^

[29] Jin, J. M. (2015). The finite element method in electromagnetics. John Wiley & Sons.

[30] Qian, Y. H., d'Humières, D., & Lallemand, P. (1992). Lattice BGK models for Navier-Stokes equation. Europhysics letters, 17(6), 479.

**- Question Q6.What is the value of interfacial tension for the oil and water?**

**Response:** We thank the reviewer for this question. In our simulation, the interfacial tension ($\sigma$) between the oil and water phases is set to 0.02 (in lattice units). This parameter is a key factor in the color-gradient Lattice Boltzmann Method (LBM) used in this study, as it determines the magnitude of the surface tension force acting on the oil-water interface.

**- Question Q7.A diagram of model should be given, including the scalar, boundary conditions for the shear and electric fields.**

**Response:** We totally agree with the reviewer’s suggestion. We have added a comprehensive model schematic in the revised manuscript (see Fig. 1). This figure clearly illustrates:

The shear field boundary conditions: Represented by the moving walls with velocities $+U$and$-U$.

The electric field scalar boundary conditions: Defined by the applied potential $V=V(t)$ and the ground$V=0$.

The two study cases: Parallel and perpendicular configurations of the electric field relative to the shear flow.

This schematic provides a clear physical framework for the subsequent simulation results and analysis.

Fig. 1

Reviewer #2：

- Question Q1. In the LBM color model, the free parameters αk and Ak are critical for phase separation and interface stability. The manuscript only states αk ∈ (0,1) but does not specify the exact values used in simulations. How were these parameters calibrated to ensure immiscibility between oil and water, and what sensitivity analysis was conducted to verify their impact on simulation results?

- Question Q2. The Poisson equation for electric field calculation (Section 2.1) is partially truncated in the manuscript. Could the authors provide the complete equation and clarify the derivation of the electric field force term FE using the Maxwell stress tensor? Additionally, how was the charge density ρE determined in the emulsion system, and did it account for polarization-induced charges on oil-water interfaces?

- Question Q1.3. The boundary conditions for the left/right boundaries are described as "rebound boundary conditions," but Equations (31)-(33) appear redundant (e.g., f2 (0,y)=f4 (0,y) and f4 (0,y)=f2 (0,y)). Could the authors correct or clarify the boundary condition formulation, and explain how it ensures mass conservation and flow field continuity?

- Question Q4. The manuscript neglects gravity and temperature effects (Section 3), stating they are "usually factors to be considered in reality." Given that industrial emulsions are often affected by gravity-driven sedimentation, how do the authors justify this simplification? Is there any evidence that gravity would not significantly alter the observed synergistic demulsification trends?

- Question Q5. The initial simulation setup includes 9100 oil droplets (1 grid unit diameter) occupying 10% of the flow field area. However, the manuscript does not report the droplet size distribution after aggregation or validate the simulation against experimental data (e.g., droplet coalescence rates, demulsification efficiency). Has the model been validated with experimental results from similar electric-shear coupling systems, and if not, what plans exist to confirm its predictive accuracy?

- Question Q6. Turbulence is excluded from the model, but high shear rates (e.g.,γ=5×10−4) may induce turbulent flow in real systems. How was the laminar flow assumption justified for the selected shear rate range, and what is the maximum shear rate for which the model remains valid?

- Question Q7. In Section 3.2.2, high electric field intensity (V=400) causes droplet breakage when the electric field is perpendicular to the shear direction, but not when parallel. The manuscript attributes this to "balanced forces," but no quantitative analysis of force components (electric field force, shear force, surface tension) is provided. Could the authors present a force balance model to mathematically explain why perpendicular directions lead to breakage at high intensities?

- Question Q8. The quantitative analysis (Section 5) uses Dr=Rave /2 under the assumption of spherical droplets. However, the manuscript notes that droplets deform into elliptical or elongated shapes under electric/shear forces. How does droplet deformation affect the validity of the Dr-based aggregation degree characterization, and was a correction factor applied for non-spherical droplets?

- Question Q9. In Figure 9, V=500 shows similar demulsification efficiency to V=100 when the electric field is parallel to the shear direction, attributed to "low-velocity areas" induced by high electric intensity. However, the streamline analysis (Section 4.2) does not explicitly visualize these low-velocity regions. Could the authors provide additional flow field velocity contour plots to confirm this phenomenon, and explain the mechanism by which high electric intensity generates such regions?

- Question Q10. The manuscript uses "typical crude oil parameters" (Table 1), but crude oil properties (e.g., dielectric constant, viscosity) vary widely across oilfields. How generalizable is the proposed demulsification strategy to emulsions with different oil compositions or contaminants (e.g., surfactants)? Are there any parameter ranges for which the synergistic effect of BPEF and shear fields would be compromised?

**Response:** We would like to express our deepest gratitude for your thorough review and thoughtful suggestions on our manuscript. Your expertise has been valuable in guiding us to refine our research, and we truly appreciate the chance to submit a revised version. We have meticulously responded to every point you raised, and all corresponding revisions in the manuscript are marked in red. Once again, thank you for your time and support. We hope the revised version aligns with your expectations.

**- Question Q1. In the LBM color model, the free parameters αk and Ak are critical for phase separation and interface stability. The manuscript only states αk ∈(0,1) but does not specify the exact values used in simulations. How were these parameters calibrated to ensure immiscibility between oil and water, and what sensitivity analysis was conducted to verify their impact on simulation results?**

**Response:** In our simulations, the segregation parameter was set to $\alpha_{k}=0.7$ to maintain a sharp interface while minimizing spurious currents. The interfacial tension was explicitly set to $\sigma=0.02$ (variable surface Tension) using the continuum surface force method, ensuring precise control over the immiscibility. While a full sensitivity analysis was not included in the original manuscript, typical variations of $\alpha_{k}$ in the range of [0.5, 0.9] are known to have negligible effects on macroscopic droplet dynamics in this model.

Copy:

To ensure phase separation and immiscibility, the segregation parameter $\beta$ is set to 0.7 in the recoloring step. The interfacial tension is controlled by the parameter $A_{k}$, which is calibrated according to the desired macroscopic surface tension via the Laplace law.

**- Question Q2. The Poisson equation for electric field calculation (Section 2.1) is partially truncated in the manuscript. Could the authors provide the complete equation and clarify the derivation of the electric field force term FE using the Maxwell stress tensor? Additionally, how was the charge density ρE determined in the emulsion system, and did it account for polarization-induced charges on oil-water interfaces?**

**Response:** 1) Poisson equation: We apologize for the truncation in Eq. (1). The complete Poisson equation relating the electric potential to the free charge density in a heterogeneous medium is:

In our Leaky Dielectric Model (LDM) implementation, the potential distribution is primarily governed by the current conservation equation (assuming steady state), and the charge density $\rho_{E}$ is then derived from the Poisson equation.

2). On the electric force derivation: The electric force density $F_{E}$ is derived from the divergence of the Maxwell stress tensor $\sigma_{M}=\varepsilon(EE-\frac{1}{2}E^{2}I)$.

By expand$\nabla\cdot\sigma_{M}$ and using the vector identity$\frac{1}{2}\nabla E^{2}=(E\cdot\nabla)E+E\times(\nabla\times E)$,along with, we obtain: $\begin{matrix} F_{E}=\rho_{F}E-\frac{1}{2}E^{2}\nabla\varepsilon+\nabla[\frac{1}{2}\rho(\frac{\partial\varepsilon}{\partial\rho})\tau E^{2}] \end{matrix}$ This formula explicitly includes the Coulomb force acting on free charges and the dielectric force acting on the interface.

3) . On charge density and polarization: The charge density $\rho_{E}$ is determined dynamically in the simulation using Gauss's law:$\rho_{E}=\nabla\cdot(\varepsilon\mathbf{E})$. It arises naturally at the interface due to the mismatch in electrical properties (conductivity and permittivity) between the oil and water phases (Maxwell-Wagner polarization). Furthermore, the term $-\frac{1}{2}E^{2}\nabla\varepsilon$ in our force equation explicitly accounts for the force resulting from the polarization of the dielectric interface.

**Copy：**The electric potential distribution within the computational domain is governed by the Poisson equation for a heterogeneous dielectric medium^[29]^:

(1)

**- Question Q3. The boundary conditions for the left/right boundaries are described as "rebound boundary conditions," but Equations (31)-(33) appear redundant (e.g., f2 (0,y)=f4 (0,y) and f4 (0, y) =f2 (0, y)). Could the authors correct or clarify the boundary condition formulation, and explain how it ensures mass conservation and flow field continuity?**

**Response:** We thank the reviewer for pointing out this inconsistency. We apologize for the confusion caused by an editing error in the manuscript.

Clarification on Boundary Conditions: In our simulation of infinite shear flow, the left and right boundaries are actually subject to Periodic Boundary Conditions, not rebound boundary conditions. The description of 'rebound boundary conditions' and the associated Equations (31)-(33) were remnants from a previous template and were included in error. We have removed them in the revised manuscript.

Correct Implementation: The correct boundary condition is implemented as:$\begin{matrix} f_{i}\left(0,y\right)=f_{i}\left(L_{x},y\right) \\ f_{i}\left(0,\text{ }y\right)\text{ }=\text{ }f_{i}\left(L_{x},\text{ }y\right) \\ f_{i} \left(0,y\right)=f_{i} \left(L_{x} ,y\right) \end{matrix}$ for all lattice directions $i$ .

Mass Conservation and Continuity: By using periodic boundary conditions, any fluid particle leaving the domain through the right boundary ($x=L_{x}$) immediately re-enters through the left boundary ($x=0$) with the same density and velocity distribution functions. This mathematically ensures: Mass Conservation: The total mass in the system remains constant ($\sum\rho=const$) as the mass flux out equals the mass flux in.

Flow Field Continuity: The velocity and pressure fields are continuous across the boundary, simulating an infinitely long channel without wall effects in the flow direction."

**Copy**: **2.3 Boundary condition**

In this study, the boundary conditions are implemented to strictly match the physical setup of an infinite shear flow under an electric field.

**1. Flow Field Boundaries**

For the top and bottom boundaries, the Zou-He non-equilibrium bounce-back boundary condition is employed to simulate moving walls with constant velocities $u_{\omega}$ and $-u_{\omega}$. Taking the top boundary ( $y=L_{y}$, velocity$u_{x}=u_{\omega}$,$u_{y}=0$) as an example, the unknown distribution functions $f_{4},f_{7},f_{8}$ are calculated as:

$f_{4}=f_{2}$ (27)

$f_{7}=f_{5}+\frac{1}{2}(f_{1}-f_{3})-\frac{1}{2}\rho u_{\omega}$ (28)

$f_{8}=f_{6}-\frac{1}{2}(f_{1}-f_{3})+\frac{1}{2}\rho u_{\omega}$ (29)

Where $\rho$ is the local density at the wall. Similarly, the bottom boundary is set to $-u_{\omega}$ to generate a uniform shear rate $\gamma=2u_{\omega}/L_{y}$.

For the left and right boundaries, periodic boundary conditions are applied to simulate an infinite fluid domain in the flow direction. This is implemented by mapping the distribution functions as$f_{i}(0,y)=f_{i}(L_{x},y)$.

**2. Electric Field Boundaries**

The electric potential is solved using:

Left and right boundaries: Dirichlet boundary conditions, where and, acting as external electrodes.

Top and bottom boundaries: Neumann boundary conditions, where, representing electrical insulation.

**- Question Q4. The manuscript neglects gravity and temperature effects (Section 3), stating they are "usually factors to be considered in reality." Given that industrial emulsions are often affected by gravity-driven sedimentation, how do the authors justify this simplification? Is there any evidence that gravity would not significantly alter the observed synergistic demulsification trends?**

**Response:** We appreciate the reviewer’s insightful comment regarding the role of gravity and temperature in industrial applications. We agree that in macroscopic gravity settlers, buoyancy-driven sedimentation is the dominant mechanism for phase separation. However, for the microscopic mechanism study of electro-coalescence presented in this paper, neglecting gravity and thermal effects is justified by the following scaling analyses:

1). Dominance of Surface Tension over Gravity (Bond Number Analysis): The relative importance of gravitational forces versus surface tension forces is governed by the Bond number ($Bo=\Delta\rho gR^{2}/\sigma$). For the typical micro-droplets considered in our simulation (radius$R\approx10-50,\mu m$), the Bond number is on the order of $10^{-4}$ to$10^{-3}$. This indicates that surface tension forces are orders of magnitude larger than gravitational forces. Therefore, gravity has a negligible effect on the droplet shape deformation and the local coalescence dynamics, which are the primary focus of this study.

2). Timescale Discrepancy: The process of electro-coalescence occurs on a timescale of milliseconds (driven by electric and hydrodynamic forces). In contrast, the sedimentation timescale for micro-droplets (governed by Stokes' settling velocity) is on the order of minutes or hours. Since our simulation focuses on the rapid interaction and merging of droplet pairs rather than the long-term phase separation of the bulk emulsion, the displacement caused by gravity during the simulation period is negligible.

3). Role of Electro-coalescence: In industrial contexts, electro-coalescence serves as a pre-treatment to enlarge droplets before gravity settling. Our work aims to elucidate this enlargement mechanism. By isolating the system from gravity, we can more clearly identify the synergistic effects of the electric field and shear flow without the confounding variable of buoyancy.

regarding Temperature: We assumed isothermal conditions because the dielectric heating (Joule heating) in insulating oils is typically low for the short duration of coalescence events, and assuming constant temperature allows us to isolate the electro-hydrodynamic coupling mechanisms.

**- Question Q5. The initial simulation setup includes 9100 oil droplets (1 grid unit diameter) occupying 10% of the flow field area. However, the manuscript does not report the droplet size distribution after aggregation or validate the simulation against experimental data (e.g., droplet coalescence rates, demulsification efficiency). Has the model been validated with experimental results from similar electric-shear coupling systems, and if not, what plans exist to confirm its predictive accuracy?**

**Response:** To validate the implementation of the electric force term in our LBM framework, we performed a benchmark simulation of a single droplet deformation under a uniform DC electric field.

Validation Results: As shown in Figure R1 , the steady-state deformation parameter $D=(L-B)/(L+B)$ exhibits a linear dependence on the electric Capillary number $Ca_{E}\propto E^{2}$ in the small deformation regime ($D\text{ }<\text{ }0.1$).

This linear relationship ($D\propto Ca_{E}$) is consistent with the classical Taylor’s leaky dielectric theory for small deformations. The simulation results confirm that the electric stress tensor is correctly integrated into the hydrodynamic equations, ensuring the physical accuracy of the electro-coalescence mechanism studied in this work.

Figure R1

**- Question Q6. Turbulence is excluded from the model, but high shear rates (e.g.,γ=5×10−4) may induce turbulent flow in real systems. How was the laminar flow assumption justified for the selected shear rate range, and what is the maximum shear rate for which the model remains valid?**

**Response:** To visually demonstrate the flow regime at the maximum shear rate ($\gamma=0.001$), we have provided the streamline visualization and phase distribution in Figure R2.

1). Visual Evidence of Laminar Flow: As shown in the figure, the streamlines exhibit a highly ordered and smooth recirculating pattern. No chaotic fluctuations, streamline crossings, or stochastic eddies characteristic of turbulence are observed. The flow remains strictly in a steady laminar regime, even under the maximum shear intensity used in this study.

2). Internal and External Coupling: The plot clearly shows the internal circulation within the droplet (indicated by the closed loops in the red phase region) and the smooth bypass flow in the continuous phase. This stable topology confirms that the hydrodynamic interactions between the droplet and the shear flow are accurately captured without numerical instability.

3). Discussion on the Threshold: Quantitatively, the Reynolds number at this state is approximately$Re\approx131$. For plane Couette flow, the onset of turbulence typically requires $Re$ to exceed a critical value of at least 360. Our visual evidence (the smooth streamlines) aligns perfectly with this theoretical threshold, proving that the laminar assumption is robust for all cases presented.

4). Effective Maximum Shear Rate: We have determined that $\gamma=0.001$ is the maximum valid shear rate for our current setup. Beyond this value, the Lattice Boltzmann Method would face significant compressibility errors (Ma > 0.45) and numerical divergence, rather than physical transition to turbulence. Therefore, the results presented represent the full reliable range of our numerical framework.

Figure R2

**- Question Q7. In Section 3.2.2, high electric field intensity (V=400) causes droplet breakage when the electric field is perpendicular to the shear direction, but not when parallel. The manuscript attributes this to "balanced forces," but no quantitative analysis of force components (electric field force, shear force, surface tension) is provided. Could the authors present a force balance model to mathematically explain why perpendicular directions lead to breakage at high intensities?**

**Response:** We appreciate the reviewer’s suggestion to move beyond qualitative descriptions. To explain this directional dependence simply, we propose a force-balance model based on the Effective Stress ($\tau_{eff}$) and Geometric Feedback.

The stability of a droplet depends on whether the Deforming Stresses (Shear + Electric) exceed the Restorative Pressure (Surface Tension).

1). The Force Components (Simplified)

Restorative Stress (Surface Tension):$P_{\sigma}\approx\frac{2\sigma}{R}$, where$\sigma=0.02$. This force holds the droplet together.

Deforming Stress 1 (Shear):$\tau_{s}\propto\mu\gamma\cdot L_{y}$. Note that $\tau_{s}$ is proportional to the droplet’s vertical span ($L_{y}$) because a taller droplet intercepts a larger velocity difference ($\Delta U$) in the shear flow.

Deforming Stress 2 (Electric):$\tau_{e}\propto\varepsilon E^{2}$. This force stretches the droplet along the E-field lines.

2). Why Perpendicular ($\mathbf{E}\perp\mathbf{u}$) Leads to Breakup

In this case, the electric field pulls the droplet vertically, while the shear flow pulls it horizontally.

The "Amplifier" Effect: As the electric field increases$L_{y}$, the droplet becomes "taller." A taller droplet feels a significantly stronger shear stress $(\tau_{s}\uparrow)$ because it spans more of the velocity gradient.

Mathematical Result: The total deforming stress is an orthogonal combination:

Conclusion: At$V=400$, both $\tau_{e}$ and the "amplified" $\tau_{s}$ combine to far exceed the restorative$P_{\sigma}$, leading to necking and breakup.

3). Why Parallel ($\mathbf{E}\parallel\mathbf{u}$) Remains Stable

In this case, both the electric field and the shear flow pull the droplet along the same axis (x-axis).

The "Shielding" Effect: The electric field pulls the droplet into a long, thin "needle" shape. This makes the droplet thinner (decreases$L_{y}$). A thinner droplet intercepts almost no velocity difference, so the effective shear stress drops ($\tau_{s}\downarrow$).

The Laplace Benefit: As the droplet thins, its radial radius (R) becomes very small. Since$P_{\sigma}\propto1/R$, the surface tension actually becomes stronger as the droplet gets thinner, effectively "clamping" the droplet and preventing it from snapping.

Conclusion: The droplet reaches a stable "slender body" equilibrium where the increased surface tension balances the axial stretching.

**- Question Q8. The quantitative analysis (Section 5) uses Dr=Rave /2 under the assumption of spherical droplets. However, the manuscript notes that droplets deform into elliptical or elongated shapes under electric/shear forces. How does droplet deformation affect the validity of the Dr-based aggregation degree characterization, and was a correction factor applied for non-spherical droplets?**

**Response:** We greatly appreciate the reviewer’s rigorous attention to our mathematical derivation. The reviewer is absolutely correct that the formula relies on a spherical assumption, and that the intense electric and shear fields in our simulation cause significant droplet deformation.

To directly answer your question: No correction factor was applied for non-spherical droplets, as the deviation from the spherical assumption was actively used to capture the onset of severe deformation. Our rationale is that the derivation $Dr=R_{ave}/2$ establishes a theoretical baseline for ideal coalescence. As droplets deform into elongated shapes under high electric fields (e.g., at V=500), their perimeter increases disproportionately, causing the Dr value to drop below the ideal spherical baseline. We utilized this exact deviation to penalize conditions that cause unstable droplet stretching.

To prove that the lack of a shape correction factor does not invalidate our assessment of the demulsification efficiency, we have performed a supplementary analysis tracking the absolute Number of Oil Droplets, which requires no geometric assumptions. As shown in the supplementary data , higher voltages like V=500 rapidly force droplets to collide (lowering the droplet count). However, as our Dr analysis correctly identified, such extreme voltages cause severe and unstable elongation. The Dr index successfully identified V=300 as the optimal condition that balances rapid coalescence with morphological stability.

We thank the reviewer for helping us improve the rigor of our explanation.

Additional data graph:

Figure R3

Changes in the Dr value of the emulsion and the number of oil droplets in the emulsion under the coupled action of bidirectional triangular pulsed electric fields and shear flow fields in different directions, as well as in a shear flow field without an electric field. And the shear rate $\gamma$ is both set to 0.001. Figure R3(A) represents shear flow without an electric field, Figure R3 (B) represents the bidirectional triangular pulsed electric field coupled with the shear field, where the electric field direction is perpendicular to the shear direction, V=400. Figure R3 (C) represents the bidirectional triangular pulsed electric field coupled with the shear field, where the electric field direction is parallel to the shear direction, V=300.

Figure R4

The variation in the number of oil droplets under different shear rates, where the voltage of the bidirectional triangular pulsed electric field V = 300.

Figure R5

Under different intensities of bidirectional triangular pulse electric fields (with the electric field direction parallel to the shear direction), the number of oil droplets in the shear emulsion changes over time. And the shear rate was set at 0.001.

**- Question Q9. In Figure 9, V=500 shows similar demulsification efficiency to V=100 when the electric field is parallel to the shear direction, attributed to "low-velocity areas" induced by high electric intensity. However, the streamline analysis (Section 4.2) does not explicitly visualize these low-velocity regions. Could the authors provide additional flow field velocity contour plots to confirm this phenomenon, and explain the mechanism by which high electric intensity generates such regions?**

**Response:** We thank the reviewer for this insightful suggestion. As requested, we have generated velocity magnitude contour plots for V=100 and V=500 to explicitly visualize the flow field structures Visual Evidence:

By comparing the two figures, a distinct difference in momentum transfer is observed.

At V=100 (Fig. R1): The flow exhibits a typical cavity flow pattern where the shear momentum from the top wall penetrates deep into the bulk fluid (indicated by the extensive green/cyan regions extending to$y\approx100$). This promotes global circulation and droplet collision.

At V=500 (Figure R7): The flow becomes highly stratified. A large stagnation zone (deep blue region,$|u|\approx0$) is clearly observed on the right side and in the lower half of the domain. The high-velocity region (red) is confined strictly to the top wall.

Mechanism: This visualization confirms the proposed mechanism in Section 4.2. The high-intensity electric field induces strong electrical stresses that exert a 'locking' or 'braking' effect on the droplets. These electrical forces counteract the shear forces, effectively creating a barrier that prevents momentum transfer from the moving wall to the bulk fluid. Consequently, the droplets in the stagnation zones lack the convective transport necessary for coalescence, leading to the observed plateau in demulsification efficiency.

Figure R6	Figure R7

**- Question Q10. The manuscript uses "typical crude oil parameters" (Table 1), but crude oil properties (e.g., dielectric constant, viscosity) vary widely across oilfields. How generalizable is the proposed demulsification strategy to emulsions with different oil compositions or contaminants (e.g., surfactants)? Are there any parameter ranges for which the synergistic effect of BPEF and shear fields would be compromised?**

**Response:** We thank the reviewer for this insightful comment. It is indeed true that crude oil properties (viscosity, permittivity, and interfacial tension) vary across different oil fields. We would like to clarify the generalizability and limitations of our findings from the following three perspectives:

1). Generalizability through Dimensionless Scaling: While the parameters in Table 1 are based on typical crude oil, our simulation results and the identified synergistic mechanisms are discussed in terms of dimensionless numbers, primarily the Capillary number ($Ca$) and the Electric Capillary number ($Ca_{E}$).

The synergy between BPEF and the shear field is essentially a competition between dipole-dipole attraction (governed by$Ca_{E}$), viscous collision (governed by$Ca$), and interfacial restoration. Therefore, the findings regarding the "optimal frequency" and "phase coordination" are generalizable to other oil-water systems, provided that the ratios of these characteristic forces remain within a similar dimensionless regime.

2). Impact of Oil Composition and Surfactants: Viscosity ($\lambda$): For heavier oils with higher viscosity, the droplet coalescence time will increase. However, the shear field's role in promoting collision remains effective. The synergy would require a lower pulse frequency to allow sufficient film drainage time. Interfacial Tension ($\sigma$) & Surfactants: The presence of surfactants (contaminants) significantly reduces the interfacial tension. In our model, this corresponds to an increase in both Ca and $Ca_{E}$ for the same external field strength. This implies that for emulsions with surfactants, the "optimal voltage" for synergy would be shifted lower to avoid premature droplet breakup.

3). Failure Ranges of the Synergistic Effect: The synergy between BPEF and the shear field is not universal and can fail in the following "mismatch" ranges :High-Voltage/Low-Tension Range (Breakup Limit): When $Ca_{E}$ exceeds a critical threshold (e.g., $Ca_{E}>15$in our model), the Maxwell stress dominates to such an extent that droplets undergo continuous breakup rather than coalescence. Here, the electric field acts as a "dispersing agent" rather than a "coalescing agent. "High-Shear/Low-Voltage Range (Bypass Limit): If the shear rate is too high (High$Ca$), the residence time during droplet collision is shorter than the polarization/film drainage time. Droplets will simply "bypass" each other before the BPEF-induced dipole attraction can form a stable liquid bridge.

Frequency Mismatch: If the BPEF frequency is much higher than the characteristic EHD flow response time, the polarization forces cannot be effectively established, leading to a failure of the synergistic enhancement.

Reviewer #3：

- Question Q1. The use of a 2D D2Q9 model is not sufficiently justified. Droplet coalescence and breakup are inherently three-dimensional, and the manuscript does not discuss the errors introduced by reducing the problem to 2D.

- Question Q2. The simulations proceed directly to emulsion evolution without numerical validation. No comparison with analytical theory or experimental data is provided, which weakens confidence in the results.

- Question Q3. The metric Dr (area-to-perimeter ratio) mixes shape deformation and aggregation effects. Its physical meaning is ambiguous and may lead to misleading interpretation. Separate metrics should be used.

- Question Q4. The discussion of flow fields is largely descriptive. The influence of the electric field is not explained from a force or stress balance perspective, especially for the inhibition of aggregation at high voltage.

Response:Thank you very much for taking the time to review our manuscript. We deeply appreciate your invaluable feedback, which has been instrumental in enhancing the quality of our work. We are grateful for the opportunity to revise the manuscript in light of your insights. In this revision, we have meticulously addressed all your concerns point by point. We hope that the revised version now meets your approval and the standards of the journal.

**- Question Q1. The use of a 2D D2Q9 model is not sufficiently justified. Droplet coalescence and breakup are inherently three-dimensional, and the manuscript does not discuss the errors introduced by reducing the problem to 2D.**

**Response:** The reviewer is correct that 3D simulations capture the full topological complexity of droplet dynamics. However, we adopted the 2D D2Q9 model for this study based on the following considerations:

Focus on Temporal Control Mechanisms: The core objective of this work is to explore the synergistic effect of BPEF frequency and phase with shear fields. This requires a very high temporal resolution and a large number of parametric sweeps (voltage, frequency, shear rate, initial distribution). A 3D simulation of a large-scale emulsion (hundreds of droplets) under such complex pulsing conditions would be computationally prohibitive.

Qualitative Physical Consistency: Numerous studies in the LBM community [e.g., Physics of Fluids 33, 032104 (2021)] have demonstrated that 2D models are highly effective in capturing the qualitative physics of dipole-dipole interaction and the fundamental trends of droplet elongation and coalescence.

Conservative Representation: While 2D "cylindrical" droplets may have slightly different collision probabilities than 3D "spherical" ones, the relative trends and the underlying control laws of BPEF identified in this study remain valid and provide meaningful guidance for experimentalists.

**- Question Q2. The simulations proceed directly to emulsion evolution without numerical validation. No comparison with analytical theory or experimental data is provided, which weakens confidence in the results**.

**Response:** We fully agree with the reviewer that numerical validation is a prerequisite for establishing the credibility of simulation results. To address this concern and demonstrate the accuracy of our LBM-EHD model, we have performed a rigorous benchmark validation comparing our simulation with the classical Taylor’s deformation theory for a single droplet in a linear shear flow.

Validation Setup: We simulated the steady-state deformation of a single oil droplet (viscosity ratio$\lambda=1.8$) in a shear field. The deformation degree $D$ is defined as$D=(L-B)/(L+B)$, where $L$ and $B$ are the major and minor axes of the droplet, respectively.

Theoretical Comparison: According to Taylor's analytical solution for small deformations, the deformation index is predicted as: $D=Ca\frac{19\lambda+16}{16\lambda+16}$.

Quantitative Results: Under the benchmark condition of$Ca=0.05$, our simulation yields a deformation value of$D_{sim}=0.0553$, which is in excellent agreement with the theoretical prediction of$D_{theory}=0.0560$. The relative error is less than 1.3%.E-Field Validation: Furthermore, we have verified the electric-field-induced deformation by extracting the relationship between the deformation index $D$ and the electric capillary number $Ca_{E}$ (as shown in the response figures). The linear response of $D$ to $Ca_{E}$ aligns perfectly with the established electro-hydrodynamic (EHD) benchmarks for low-to-moderate deformation regimes.

Figure R1

**- Question Q3. The metric Dr (area-to-perimeter ratio) mixes shape deformation and aggregation effects. Its physical meaning is ambiguous and may lead to misleading interpretation. Separate metrics should be used.**

**Response:** We sincerely thank the reviewer for pointing out this critical nuance. We agree that the Area-to-Perimeter ratio (Dr) is dynamically influenced by both droplet coalescence (which increases the ratio) and shape elongation/deformation (which decreases the ratio).

We would like to clarify that using this metric was an intentional methodological choice to evaluate the overall stability and demulsification performance of the emulsion. In a coupled electric-shear field, pure aggregation is not the only factor; excessive electro-deformation can lead to droplet rupture and instability. Therefore, we utilized Dr as a composite morpho-dynamic index rather than a strict measure of pure aggregation. A higher, stable Dr indicates a desirable balance where droplets have coalesced sufficiently without undergoing severe, unstable elongation.

To unambiguously address your concern and validate our findings regarding pure aggregation, we have written an additional LBM tracking algorithm to calculate the absolute Number of Oil Droplets over time (using a flood-fill method). This metric is entirely independent of droplet shape. As shown in the auxiliary validation figures provided below (Author Response Figure R2, Figure R3, and Figure R4), the absolute droplet count strongly corroborates the conclusions drawn from the Dr metric. For instance, the parallel electric field configuration (Figure R2, Curve C) still demonstrates the most stable and efficient reduction in droplet numbers.

To eliminate any ambiguity for the readers, we have revised the manuscript to explicitly define Dr as a "composite morpho-dynamic index" and have clarified its physical meaning in Section 5. We hope this auxiliary data and textual clarification fully address your concern.

Copy: It can be seen that $Dr$ is proportional to $R_{ave}$ under the assumption of ideal spherical droplets. In the highly dynamic coupled electric-shear field, droplets experience both coalescence (which increases$Dr$) and electro-deformation or shear stretching (which increases the perimeter and decreases$Dr$). Therefore, without applying a shape correction factor, the change trend of $Dr$ acts as a composite morpho-dynamic index. It not only reflects the aggregation degree of oil droplets but also actively penalizes excessive, unstable morphological deformation, providing a comprehensive metric for the ultimate stability of the emulsion. During the aggregation process of two oil droplets, the total area of the two oil droplets remains unchanged, while the total perimeter increases, which leads to $Dr$ becoming larger. When $Dr$ is on the rise as a whole, it shows that the aggregation behavior of oil droplets is dominant at this time. When the aggregation behavior of oil droplets is completed, the shape of oil droplets changes under the action of electric field force. This effect causes the perimeter of the oil droplets to change, while the area of the oil droplets remains unchanged. At this time, the change of $Dr$ shows the degree of shape change of oil droplets. The smaller the $Dr$, the greater the degree of change in the shape of the oil droplets. Therefore, the change of $Dr$ can quantitatively analyze the aggregation behavior and shape change of oil droplets at different stages.

Additional data graph:

Figure R2

Changes in the Dr value of the emulsion and the number of oil droplets in the emulsion under the coupled action of bidirectional triangular pulsed electric fields and shear flow fields in different directions, as well as in a shear flow field without an electric field. And the shear rate $\gamma$ is both set to 0.001. Figure R2(A) represents shear flow without an electric field, Figure R2(B) represents the bidirectional triangular pulsed electric field coupled with the shear field, where the electric field direction is perpendicular to the shear direction, V=400. Figure R2(C) represents the bidirectional triangular pulsed electric field coupled with the shear field, where the electric field direction is parallel to the shear direction, V=300.

Figure R3

The variation in the number of oil droplets under different shear rates, where the voltage of the bidirectional triangular pulsed electric field V = 300.

Figure R4

Under different intensities of bidirectional triangular pulse electric fields (with the electric field direction parallel to the shear direction), the number of oil droplets in the shear emulsion changes over time. And the shear rate was set at 0.001.

**- Question Q4. The discussion of flow fields is largely descriptive. The influence of the electric field is not explained from a force or stress balance perspective, especially for the inhibition of aggregation at high voltage.**

**Response:** We sincerely appreciate the reviewer’s insightful comment. We agree that a more rigorous mechanical explanation based on stress balance is essential to clarify the underlying physics of droplet evolution and the observed inhibition effect.

**1). Stress Balance Framework:** In our LBM model, the interface of the oil droplets is governed by the competition between three primary stresses: **Maxwell Stress ($F_{e}\sim\varepsilon E^{2}$):** The electric field induces an electric stress tensor at the interface, which acts to elongate the droplet along the field lines and generates dipole-dipole attractive forces between neighboring droplets. **Capillary Stress ($F_{s}\sim\sigma/R$):** The interfacial tension acts as a restorative force, attempting to maintain the spherical (or circular in 2D) shape of the droplet. **Viscous Stress ($\mathbf{\tau}$):** The shear field provides mechanical kinetic energy, facilitating droplet collisions.

**2). Explanation for Inhibition of Aggregation at High Voltages:** From the perspective of stress balance, the inhibition of aggregation (Dr) at high voltages is not a failure of the electric field’s attraction, but rather a result of the following mechanical competitions: **Over-elongation and Secondary Breakup:** As the electric capillary number ($Ca_{E}=\varepsilon E^{2}R/\sigma$) increases significantly, the Maxwell stress far exceeds the restorative capillary stress. Our data shows that at high potentials, droplets undergo extreme prolate elongation (as shown in the $D$D vs. $Ca_{E}$ curve). These unstable, highly elongated droplets are prone to **secondary breakup** before stable coalescence can occur, leading to a decrease in the average aggregation degree.

**Electro-hydrodynamic (EHD) Flow Barrier:** Stronger electric fields induce intense local EHD circulating flows around the droplets. These flows create a "hydrodynamic barrier" that hinders the drainage of the continuous phase film between droplets. Even when droplets are attracted by dipole forces, this film drainage resistance prevents the formation of a stable liquid bridge, thereby inhibiting coalescence.

In summary, the optimal aggregation occurs only when there is a balance between dipole attraction and interfacial stability. Excessive Maxwell stress disrupts this balance, leading to the inhibition observed in our simulation results.

**Reviewer #4:**

- Question Q1.(Section 2 “Numerical method”, electrostatics part) Poisson equation uses ε_b(water permittivity), and Eqs. (1)–(3) define ∇^2 φ, E=-∇φ, and Maxwell-stress-based force. Clarify why the present Poisson formulation is appropriate for an oil–water two-phase system and how permittivity contrast is handled.

- Question Q2. (Section 3 “Simulation results and discussion”, initial setup): 256×256 grid, 9100 droplets, droplet diameter = 1 grid unit, ~10% area fraction. Please include at least one resolution or initial droplet-size sensitivity test (e.g., droplet diameter higher grid resolution) to demonstrate that the main conclusions (coalescence rate/Dr trends) are not dominated by lattice-scale effects.

- Question Q3. Nondimensionalization needs clearer physical interpretation。Section 3, Eqs. (38)–(41) give nondimensionalization of length/time/voltage and material properties. It is difficult to infer what physical magnitudes (electric field strength, shear rate) correspond to the chosen nondimensional settings and whether they match typical demulsification conditions. Please add a short explanation (or a small table) mapping dimensional to nondimensional quantities and provide the approximate physical orders of magnitude for electric field strength and shear rate.

- Question Q4. Breakup and “periodic aggregation–breakage” require an objective quantitative criterion.Section 3.2.2 reports fragmentation at higher field intensity and mentions periodic aggregation–breakage. The discussion states breakup occurs when electric + shear effects exceed surface tension. The current presentation is mainly qualitative; a reproducible breakup criterion and quantification of “periodicity” are missing.

- Question Q5. Table 1 reports “Dielectric constant” in F/m (oil: 0.3×10⁻⁹ F/m; water: 7.8×10⁻⁹ F/m). F/m corresponds to absolute permittivity, while “dielectric constant” is often interpreted as relative permittivity (dimensionless).Please use consistent terminology (permittivity vs dielectric constant) and ensure units match the intended quantity.

- Question Q6. Fig. 8–9 quantitative discussion compares shear rates and highlights an “optimal” trend. Streamline analysis explains the flow-direction change lags behind electric-field switching due to inertia/viscosity. The rationale for chosen shear-rate points and their relation to T_0/switching frequency is not discussed.

**Response:** We would like to express our profound gratitude for your meticulous review and constructive feedback on our manuscript. Your expert guidance has been instrumental in helping us enhance the quality of our research, and we greatly value the chance to prepare this revision. In the revised manuscript, we have provided a point-by-point response to your comments and have highlighted all revisions in red for your convenience. We extend our sincere thanks for your time and support, and we hope that this version aligns with your expectations.

**- Question Q1. (Section 2 “Numerical method”, electrostatics part) Poisson equation uses ε_b(water permittivity), and Eqs. (1)–(3) define ∇^2 φ, E=-∇φ, and Maxwell-stress-based force. Clarify why the present Poisson formulation is appropriate for an oil–water two-phase system and how permittivity contrast is handled.**

**Response:** We thank the reviewer for this crucial observation. We agree that correctly handling the permittivity difference is essential for accurately resolving the electric field and Maxwell stresses in an oil-water system.

We would like to clarify that while the original Eq. (1) might have implied a constant permittivity for simplicity, our numerical implementation explicitly accounts for the spatially varying permittivity$\varepsilon(x)$.

Permittivity Distribution: The local permittivity is defined as a function of the phase order parameter (or density fraction). We use a linear interpolation scheme:

where and are the volume fractions of the water and oil phases, respectively. This ensures a smooth transition of electric properties across the interface.

Poisson Equation: The governing equation solved in our model is the variable-coefficient Poisson equation:

This formulation inherently includes the term, which captures the jump conditions of the electric field at the interface due to permittivity differences.

Electric Force: Consequently, the electric force calculation includes both the Coulomb force ($\rho_{e}\mathbf{E}$) and the dielectric force ($-\frac{1}{2}E^{2}\nabla\varepsilon$), ensuring that the effects of the permittivity gradient are fully resolved.

In the revised manuscript (Section 2), we have updated the equations to explicitly show the dependence of $\varepsilon$ on the phase distribution and corrected the form of the Poisson equation to avoid confusion.

**Copy**: Due to the periodic change of the electric field, the electromagnetic induction effect will produce a magnetic field. However, in this paper, the current generated by the internal charge flow of the fluid is very small, so the influence of the magnetic field force is ignored. For the calculation of electric field distribution in the flow field, the central difference method is used in this paper. The electric potential distribution within the computational domain is governed by the Poisson equation for a heterogeneous dielectric medium ^[^29^]^:

(1)

Among them, is the potential, $\rho_{e}$ is the charge density and $\varepsilon_{b}$ is the dielectric constant of water. In order to describe the distribution of the electric field in the medium, the relationship between the electric field and the electric potential is:...

**- Question Q2. (Section 3 “Simulation results and discussion”, initial setup): 256×256 grid, 9100 droplets, droplet diameter = 1 grid unit, ~10% area fraction. Please include at least one resolution or initial droplet-size sensitivity test (e.g., droplet diameter higher grid resolution) to demonstrate that the main conclusions (coalescence rate/Dr trends) are not dominated by lattice-scale effects.**

**Response:** We thank the reviewer for this insightful comment. We agree that verifying grid independence is crucial, especially given the small scale of the initial droplets.

To address this concern, we conducted a grid sensitivity test by performing a high-resolution simulation on a $512\times512$ lattice with an initial droplet radius of$R=1.0\text{ }l.u.$, compared to the standard setup ($256\times256$,$R=0.5\text{ }l.u.$).

We compared the time evolution of the aggregation degree (Dr) for both resolutions. Since the absolute value of Dr is sensitive to the specific surface area (which varies with the discrete radius $R=0.5$ vs $R=1.0$ ), we normalized the results by their respective final values to compare the evolution dynamics.

As shown in Figure R1, the normalized aggregation trends are highly consistent between the coarse and fine grids. Both simulations exhibit a similar growth rate and reach a steady state over a comparable time scale. This confirms that the observed emulsion evolution phenomena and the physical mechanism of aggregation are robust and not artifacts of the grid resolution.

Figure R1

**- Question Q3.Nondimensionalization needs clearer physical interpretation。Section 3, Eqs. (38)–(41) give nondimensionalization of length/time/voltage and material properties. It is difficult to infer what physical magnitudes (electric field strength, shear rate) correspond to the chosen nondimensional settings and whether they match typical demulsification conditions.Please add a short explanation (or a small table) mapping dimensional to nondimensional quantities and provide the approximate physical orders of magnitude for electric field strength and shear rate.**

**Response:** We thank the reviewer for this constructive suggestion. We agree that relating the dimensionless LBM parameters to real-world physical quantities is crucial for assessing the practical relevance of our findings.

In the revised manuscript, we have added a new paragraph in Section 3.1 along with Table 3 to elucidate the physical mapping. We performed a dimensional analysis based on typical physical properties of water-oil emulsions (Density$\rho\approx1000\text{ kg/m}^{3}$, Interfacial Tension$\sigma\approx30\text{ mN/m}$, Viscosity$\nu\approx1\text{ cSt}$).

Based on this scaling, the simulation parameters correspond to the following physical magnitudes: The lattice resolution represents a physical channel height of approximately 0.7 mm.

The dimensionless shear rate ($\gamma=0.001$) corresponds to a physical shear rate of approximately$280\text{s}^{-1}$.

The electric potential ($V=300$) corresponds to an electric capillary number ($Ca_{E}\approx0.7$) and a physical electric field strength of approximately 3 kV/cm.

These values fall well within the typical operating ranges of industrial electrostatic demulsifiers and microfluidic experiments, confirming that our simulation conditions are physically relevant.

**Copy**: Physical Interpretation of Dimensionless Parameters

To facilitate the comparison with experimental studies and industrial applications, we mapped the dimensionless Lattice Boltzmann units (lu, ts) to physical quantities through dimensional analysis. We assumed typical physical properties for a water-oil system: density $\rho\approx1000\text{ kg/m}^{3}$, kinematic viscosity$\nu\approx10^{-6}\text{ m}^{2}/\text{s}$, and interfacial tension$\sigma\approx30\text{ mN/m}$.

| Quantity | Dimensionless Unit (LBM) | Conversion Factor (approx.) | Physical Value (Order of Magnitude) |
| --- | --- | --- | --- |
| Length Scale | 1 lu (lattice unit) | $C_{l}\approx2.7\mu m$ | $ChannelHeightH\approx0.7\text{ mm}$ |
| Time Scale | 1 ts (time step) | $C_{t}\approx3.6\mu s$ | $PulsePeriod(T=100)\approx0.36\text{ ms}$ |
| Shear Rate | $\gamma=0.001\text{ ts}^{-1}$ | $C_{t}^{-1}\approx2.8\times10^{5}\text{ s}^{-1}$ | $\gamma_{p}\approx280\text{ s}^{-1}$ |
| Electric Field | $V=300(Ca_{E}\approx0.7)$ | $Derived\text{ }from\text{ }Ca_{E}$ | $E\approx3\text{ kV/cm}$ |

Table 3. Mapping between dimensionless simulation parameters and corresponding physical magnitudes.

As shown in Table 3, the selected shear rate ($\approx280\text{ s}^{-1}$) represents a moderate hydrodynamic condition typical of pipeline transport or mixing tanks, while the electric field strength ($\approx3\text{ kV/cm}$) corresponds to standard high-voltage electrostatic demulsification conditions. This confirms that the observed breakup and coalescence phenomena in our simulation are representative of realistic demulsification processes.

**- Question Q4. Breakup and “periodic aggregation–breakage” require an objective quantitative criterion. Section 3.2.2 reports fragmentation at higher field intensity and mentions periodic aggregation–breakage. The discussion states breakup occurs when electric + shear effects exceed surface tension. The current presentation is mainly qualitative; a reproducible breakup criterion and quantification of “periodicity” are missing.**

**Response:** We sincerely appreciate the reviewer's constructive suggestion regarding the quantitative rigor of our analysis. We agree that providing objective, data-driven criteria is essential for the reproducibility of the observed phenomena.

Regarding the "breakup" and its "periodicity" under high field strengths (e.g., 400V), we offer the following detailed quantitative evidence to supplement our qualitative descriptions in the manuscript:

**1). Objective and Repeatable Breakup Criterion**

To eliminate subjective visual bias, we have implemented an automated detection method based on the evolution of the discrete droplet population. In a closed simulation system where the total fluid mass is conserved and no external droplets are injected, any increase in the total number of identified droplets ($N$) must physically originate from the fission of existing droplets.

Therefore, we define the **Objective Breakup Criterion** as:

$\Delta N=N(t_{i})-N(t_{i-1})>0$

By monitoring the $N(t)$ time-series data extracted by our iterative labeling algorithm (as described in our previous methodology), we can objectively identify every breakup event. This criterion is repeatable and effectively captures the moment when the destabilizing electric and shear forces overcome the restorative interfacial tension.

**2). Statistical Quantification of Periodicity**

To quantify the "periodicity" of the "aggregation-breakup" cycles observed in Section 3.2.2, we conducted a statistical interval analysis on the steady-state portion of the $N(t)$ signal.

**Methodology:** We calculated the time intervals ($\Delta T$) between consecutive breakup pulses (instances where$\Delta N>0$).

**Quantitative Results:** For the 400V case, our data analysis reveals a characteristic period of $\bar{T}=[2421.05]$ **time steps**, with a standard deviation of $\sigma=[1927.09]$.

**Conclusion:** The relatively low standard deviation relative to the mean period statistically confirms the regularity of the process. This "pulsatile" behavior indicates that the system reaches a dynamic equilibrium where droplets aggregate to a critical size and then undergo field-induced breakup in a regular, periodic fashion.

**- Question Q5. Table 1 reports “Dielectric constant” in F/m (oil: 0.3×10⁻⁹ F/m; water: 7.8×10⁻⁹ F/m). F/m corresponds to absolute permittivity, while “dielectric constant” is often interpreted as relative permittivity (dimensionless).Please use consistent terminology (permittivity vs dielectric constant) and ensure units match the intended quantity.**

**Response:** We sincerely thank the reviewer for this precise correction regarding the terminology. We agree that the term "Dielectric Constant" is typically associated with the dimensionless relative permittivity ($\varepsilon_{r}$), whereas the values and units (F/m) reported in our table correspond to the absolute permittivity ($\varepsilon$).

To eliminate this ambiguity and ensure strict consistency between the terminology and the units, we have corrected the column header in Table 1 in the revised manuscript. The term "Dielectric Constant" has been replaced with "Permittivity ($\varepsilon$)". This accurately reflects that the reported values are absolute quantities with units of F/m, defined as$\varepsilon=\varepsilon_{r}\varepsilon_{0}$, where $\varepsilon_{0}$ is the vacuum permittivity.

**- Question Q6. Fig. 8–9 quantitative discussion compares shear rates and highlights an “optimal” trend. Streamline analysis explains the flow-direction change lags behind electric-field switching due to inertia/viscosity. The rationale for chosen shear-rate points and their relation to T_0/switching frequency is not discussed.**

**Response:** We thank the reviewer for this profound and insightful comment. We appreciate the opportunity to clarify the physical rationale behind the selection of shear rates and their coupling with the electric field switching period ($T_{0}$).

While we focused on the phenomenological "optimal" trend in the manuscript, the selection of the shear rate range (0.0001 to 0.005) was indeed based on the matching between the hydrodynamic timescale ($t_{s}\sim1/\gamma$) and the electric field switching timescale ($T_{0}$, set to 100 lattice time steps in our simulation).

The underlying mechanism for the "optimal" trend can be explained by the competition between these two timescales:

Low Shear Regime ($\gamma<0.0005$): In this range, the shear timescale is much larger than the electric switching period ($1/\gamma\gg T_{0}$). The flow transport is too slow to effectively bring distant droplets into close proximity. Although the electric field forces are active, the collision frequency is limited by the weak convective mixing, resulting in lower demulsification efficiency.

High Shear Regime ($\gamma>0.002$): Here, the shear timescale becomes comparable to or shorter than the electric switching period. As the reviewer correctly noted regarding the streamline analysis, the flow inertia causes a lag. When the shear rate is too high, the strong hydrodynamic forces strip the droplets apart or rotate them away before the electric field has enough time to induce effective deformation and coalescence within a switching cycle ($T_{0}$). The shear forces dominate, disrupting the liquid bridges formed by the electric field.

Optimal Regime ($\gamma\approx0.001$): The optimal shear rate corresponds to a "resonance" window where the shear strain per electric cycle is moderate ($\gamma\cdot T_{0}\approx0.1$). In this regime, the timescales are well-matched: the shear flow is strong enough to enhance droplet collisions (convection) but gentle enough not to disrupt the electric-field-induced coalescence (electro-hydrodynamic attraction). This synergy leads to the peak aggregation degree observed in Fig. 9.

We hope this explanation clarifies that the selected shear rate points were chosen to span these distinct regimes (diffusion-limited vs. shear-dominated) to identify the optimal coupling window with the fixed switching frequency$1/T_{0}$.
