# Supplementary material for: Simulation of emulsion evolution in shear flow field under the control of bidirectional pulsed electric field
Source: PLoS One. 2026 Jul 10;21(7):e0336345. doi: 10.1371/journal.pone.0336345 (PMC13354001; doi:10.1371/journal.pone.0336345)
Supplement: Attachment — Submitted filename: response letter.docx [file pone.0336345.s005.docx]

Dear Editors and Reviewers:

We appreciate the opportunity to revise our manuscript titled " Simulation of emulsion evolution in shear flow field under the control of bidirectional pulsed electric field" and are grateful for the insightful comments provided by the reviewers. We have carefully addressed all the comments of reviewers and revised the manuscript accordingly.

**- Question Q1:** Acronyms such as LBM, BGK which appear for the first time in the text, must be defined.

**Response:** Thank you for this valuable comment. We agree that all acronyms should be clearly defined at their first occurrence.

In the revised manuscript, we have carefully checked and corrected this issue. Specifically: “LBM” has been revised to **“lattice Boltzmann method (LBM)”** at its first appearance in Introduction.

Copy: “BGK” has been revised to **“Bhatnagar–Gross–Krook (BGK)”** when first introduced in the collision model description.

All subsequent occurrences use the abbreviations consistently.

**- Question Q2:** “some scholars have found that changing the dielectric constant and conductivity can regulate droplet deformation in shear flow fields” The first word at the beginning of a sentence should be capitalised.

**Response:** Thank you for pointing out this grammatical issue.

We have corrected the sentence in the revised manuscript by capitalizing the first word. Revised as: **“Some scholars have found that changing the dielectric constant and conductivity can regulate droplet deformation in shear flow fields.”** In addition, we have conducted a thorough proofreading of the entire manuscript to ensure that all sentences begin with proper capitalization.

**- Question Q3**: The variables in the equations in Section 2.1 and subsequent sections are not accompanied by units.

**Response:** Thank you for this important comment.

We would like to clarify that the governing equations in this study are primarily expressed in a **dimensionless lattice Boltzmann framework**, where variables are normalized using lattice units (lu) and time steps (ts). Therefore, most variables in Section 2.1 do not carry conventional SI units.

To address this concern and improve clarity, we have made the following revisions:

**Supplemented Table 3**, which provides: Mapping between dimensionless parameters and their corresponding **physical magnitudes and units** (e.g., shear rate, electric field strength).

**Improved the Symbol Table**, where:

Units (SI) are now explicitly listed for each physical variable where applicable.

**- Question Q**4: the selected shear rate (≈ 280 s-1)，while the electric field strength (≈ 3 kV/cm) " Why not provide accurate figures?

**Response:** Thank you for this insightful comment.

The reported values (≈ 280 s⁻¹ and ≈ 3 kV/cm) are obtained through dimensional mapping between lattice Boltzmann units and physical quantities based on characteristic scaling (see Table 3). Due to the inherent scaling freedom of the lattice Boltzmann framework, this mapping is not unique, and therefore these values represent **order-of-magnitude estimates rather than exact physical quantities.**

To clarify this point, we have revised the manuscript as follows: The description in the main text has been modified to explicitly state that these values correspond to **typical industrial ranges** rather than precise measurements.

Table 3 has been updated to clarify that the reported values are **approximate and scaling-dependent.**

A note has been added to explain the origin and limitation of the dimensional conversion.

These revisions improve the physical interpretation and transparency of the simulation parameters.

**Question5: In reality, gravity and temperature are usually factors to be considered, which are ignored in this paper. Please provide the basis for this assumption and explain it.**

**Response:** We appreciate the reviewer’s valuable comment regarding the physical completeness of the model. In this study, we intentionally adopted an isothermal assumption and neglected gravity to isolate and clarify the synergistic mechanisms of the Bidirectional Pulse Electric Field (BPEF) and the shear field. The justifications for these assumptions are as follows:

**1. Justification for Neglecting Gravity (Bond Number Analysis):**
The relative importance of gravity versus interfacial tension can be characterized by the dimensionless **Bond number (**$Bo$**)**:

$$Bo=\frac{\Delta\rho gR^{2}}{\sigma}$$

where $\Delta\rho$ is the density difference, $g$is gravity, $R$is the droplet radius, and $\sigma$ is the interfacial tension.

In typical micro-scale emulsions (droplet size$10\sim100\mu m$), the surface tension force dominates over the gravitational/buoyancy force. For the parameters used in our study, the Bond number is much smaller than unity ($Bo\ll1$), indicating that gravity has a negligible effect on droplet deformation and short-range dipole-dipole interactions compared to Maxwell stress and capillary stress.

Therefore, neglecting gravity is a standard and justified simplification in studies focusing on the electro-hydrodynamics (EHD) of fine emulsions.

**2. Justification for the Isothermal Assumption:**

**Mechanism Isolation:** This work focuses on the **mechanical and electrical coupling** (the competition between Maxwell stress, viscous stress, and surface tension). By maintaining a constant temperature (isothermal condition), we can isolate the effects of electric field frequency and phase without the interference of thermocapillary (Marangoni) flows or temperature-dependent viscosity variations.

**Time Scale:** The characteristic time for droplet collision and coalescence under strong electric and shear fields is significantly shorter than the time scale of significant heat transfer or temperature-induced property changes in the bulk phase.

**Practicality in LBM:** In most Lattice Boltzmann Method (LBM) studies of electro-demulsification, the isothermal assumption is a standard benchmark to establish the fundamental control laws before introducing complex multi-physics thermal coupling.

**Copy: To isolate the synergistic mechanism of the bidirectional pulsed electric field (BPEF) and the shear field, this study assumes an isothermal environment and neglects the effect of gravity.**

**For micro-scale emulsions (with typical droplet sizes of** $10\sim100\mu m$**), the relative importance of gravity compared to interfacial tension is characterized by the Bond number (**$Bo=\Delta\rho gR^{2}/\sigma$**). Given the micro-scale dimensions and fluid properties, the Bond number is much smaller than unity (**$Bo\ll1$**), indicating that the droplet dynamics are completely dominated by the Maxwell stress, viscous shear, and interfacial capillary forces, making gravity negligible.**

**Furthermore, the isothermal assumption is adopted to deliberately exclude the complex interference of thermocapillary (Marangoni) flows and temperature-dependent viscosity variations, thereby allowing a focused investigation into the pure electro-hydrodynamic (EHD) coupling laws.**

**- Question 6:** In addition, the model in this paper cannot deal with the case of turbulence.” Could the author please explain whether the model studied in this paper is suited to laminar or turbulent flow conditions? If it is not suited to turbulent conditions, but the flow pattern of the emulsion becomes turbulent when the shear rate is too high, how should this be addressed?

**Response:** We thank the reviewer for pointing out this boundary condition. The current LBM-EHD model is indeed a laminar flow solver. We believe this is the appropriate regime for the physical scale and the scientific objectives of this study for the following reasons:

**1. Reynolds Number (**$\mathbf{Re}$**) at the Micro-scale:**

In our study, the characteristic length scale is the droplet diameter ($10\sim100\mu m$) and the gap between electrodes is small. The Reynolds number () remains low even at relatively high shear rates because of the small characteristic length ($L$) and the high viscosity ($\mu$) of the oil phase. In laboratory-scale electrostatic coalescers and microfluidic devices, the flow typically remains in the laminar or transitional regime rather than fully developed turbulence.

**2. Local Laminar Assumption (Kolmogorov Scale):**

Even if the global flow in an industrial-scale demulsifier were turbulent, the interaction between two microscopic droplets often occurs at scales smaller than the **Kolmogorov microscale** (the smallest dissipative scales of turbulence). At this sub-grid scale, the local relative velocity field can be effectively approximated as a **locally linear laminar shear flow**. Therefore, our model provides a high-fidelity representation of the "local" physics of coalescence that occurs within a larger turbulent system.

**3. Addressing High-Shear Turbulence:**

If the shear rate were increased to a point where the flow becomes inherently turbulent, the current model would need to be addressed in the following ways:

Numerical Adjustment: We would need to incorporate a Sub-Grid Scale (SGS) model, such as Large Eddy Simulation (LES) within the LBM framework, to account for turbulent eddy viscosity and energy dissipation.

Physical Effect: In a turbulent regime, the random fluctuations would likely overcome the structured dipole-dipole attraction of the BPEF. The "synergistic effect" we identified might weaken as the deterministic shear trajectory is replaced by stochastic turbulent collisions.

Copy: "In addition the current model assumes a laminar regime, which is consistent with the micro-scale droplet interactions studied. For industrial applications involving high-Reynolds-number turbulence, the model would require integration with Large Eddy Simulation (LES) methods to account for stochastic turbulent fluctuations."

In addition, we have included streamlines of the flow field at two higher shear rates (Figure X: 0.002 and 0.003). As shown, the flow exhibits regions of accelerated and decelerated motion and localized vortices under high shear, but no fully developed turbulence is observed. This indicates that the flow remains predominantly laminar, albeit with strong shear gradients.

It should be noted that the present lattice Boltzmann model does not incorporate turbulence modeling (e.g., LES or RANS), and therefore cannot capture turbulence-induced multi-scale droplet breakup. Consequently, the model is strictly applicable to laminar flow regimes. For flows exceeding the laminar regime, advanced LBM approaches with turbulence closure, such as LES- or MRT-based schemes, would be required.

\

Streamline diagram of the flow field at a shear rate of 0.002

Streamline diagram of the flow field at a shear rate of 0.003
