# Supplementary material for: Simulation of emulsion evolution in shear flow field under the control of bidirectional pulsed electric field
Source: PLoS One. 2026 Jul 10;21(7):e0336345. doi: 10.1371/journal.pone.0336345 (PMC13354001; doi:10.1371/journal.pone.0336345)
Supplement: Peer review history

# Peer review: Decision Letter 0

**DOI:** 10.1371/journal.pone.0336345.r001

**Authors:** P. Davide Cozzoli

5 Jan 2026

PONE-D-25-56801

Simulation of emulsion evolution in shear flow field under the control of bidirectional pulsed electric field

PLOS One

Dear Dr. Wang,

Thank you for submitting your manuscript to PLOS ONE. After careful consideration, we find that it has merit but does not meet several PLOS ONE’s publication criteria as it currently stands. Therefore, we invite you to submit a carefully revised version of the manuscript that addresses the major issues raised during the review process by 4 independent expert reviewers selected by us.

Please submit your revised manuscript by Feb 19 2026 11:59PM. If you will need more time than this to complete your revisions, please reply to this message or contact the journal office at plosone@plos.org. When you're ready to submit your revision, log on to https://www.editorialmanager.com/pone/ and select the 'Submissions Needing Revision' folder to locate your manuscript file.

Please include the following items when submitting your revised manuscript:

- A letter that responds to each point raised by the academic editor and reviewer(s). You should upload this letter as a separate file labeled 'Response to Reviewers'.
- A marked-up copy of your manuscript that highlights changes made to the original version. You should upload this as a separate file labeled 'Revised Manuscript with Track Changes'.
- An unmarked version of your revised paper without tracked changes. You should upload this as a separate file labeled 'Manuscript'.

If you would like to make changes to your financial disclosure, please include your updated statement in your cover letter. Guidelines for resubmitting your figure files are available below the reviewer comments at the end of this letter.

If applicable, we recommend that you deposit your laboratory protocols in protocols.io to enhance the reproducibility of your results. Protocols.io assigns your protocol its own identifier (DOI) so that it can be cited independently in the future. For instructions see: https://journals.plos.org/plosone/s/submission-guidelines#loc-laboratory-protocols. Additionally, PLOS ONE offers an option for publishing peer-reviewed Lab Protocol articles, which describe protocols hosted on protocols.io. Read more information on sharing protocols at https://plos.org/protocols?utm_medium=editorial-email&utm_source=authorletters&utm_campaign=protocols.

We look forward to receiving your revised manuscript.

Kind regards,

P. Davide Cozzoli

Academic Editor

PLOS One

Journal Requirements:

When submitting your revision, we need you to address these additional requirements.

1. Please ensure that your manuscript meets PLOS ONE's style requirements, including those for file naming. The PLOS ONE style templates can be found at

https://journals.plos.org/plosone/s/file?id=wjVg/PLOSOne_formatting_sample_main_body.pdf and

https://journals.plos.org/plosone/s/file?id=ba62/PLOSOne_formatting_sample_title_authors_affiliations.pdf

2. Please note that PLOS One has specific guidelines on code sharing for submissions in which author-generated code underpins the findings in the manuscript. In these cases, we expect all author-generated code to be made available without restrictions upon publication of the work. Please review our guidelines at https://journals.plos.org/plosone/s/materials-and-software-sharing#loc-sharing-code and ensure that your code is shared in a way that follows best practice and facilitates reproducibility and reuse.

3. Please update your submission to use the PLOS LaTeX template. The template and more information on our requirements for LaTeX submissions can be found at http://journals.plos.org/plosone/s/latex.

4. We note that the grant information you provided in the ‘Funding Information’ and ‘Financial Disclosure’ sections do not match.

When you resubmit, please ensure that you provide the correct grant numbers for the awards you received for your study in the ‘Funding Information’ section.

5. Thank you for stating the following financial disclosure:

[The author(s) received no specific funding for this work.].

At this time, please address the following queries:

a) Please clarify the sources of funding (financial or material support) for your study. List the grants or organizations that supported your study, including funding received from your institution.

b) State what role the funders took in the study. If the funders had no role in your study, please state: “The funders had no role in study design, data collection and analysis, decision to publish, or preparation of the manuscript.”

c) If any authors received a salary from any of your funders, please state which authors and which funders.

d) If you did not receive any funding for this study, please state: “The authors received no specific funding for this work.”

Please include your amended statements within your cover letter; we will change the online submission form on your behalf.

6. Thank you for stating the following in the Acknowledgments Section of your manuscript:

[We acknowledge the National Natural Science Foundation of China (Program NoS.12161058and 12361096).]

We note that you have provided funding information that is not currently declared in your Funding Statement. However, funding information should not appear in the Acknowledgments section or other areas of your manuscript. We will only publish funding information present in the Funding Statement section of the online submission form.

Please remove any funding-related text from the manuscript and let us know how you would like to update your Funding Statement. Currently, your Funding Statement reads as follows:

[The author(s) received no specific funding for this work.]

Please include your amended statements within your cover letter; we will change the online submission form on your behalf.

7. We note that your Data Availability Statement is currently as follows: [All relevant data are within the manuscript and its Supporting Information files.]

Please confirm at this time whether or not your submission contains all raw data required to replicate the results of your study. Authors must share the “minimal data set” for their submission. PLOS defines the minimal data set to consist of the data required to replicate all study findings reported in the article, as well as related metadata and methods (https://journals.plos.org/plosone/s/data-availability#loc-minimal-data-set-definition).

For example, authors should submit the following data:

- The values behind the means, standard deviations and other measures reported;

- The values used to build graphs;

- The points extracted from images for analysis.

Authors do not need to submit their entire data set if only a portion of the data was used in the reported study.

If your submission does not contain these data, please either upload them as Supporting Information files or deposit them to a stable, public repository and provide us with the relevant URLs, DOIs, or accession numbers. For a list of recommended repositories, please see https://journals.plos.org/plosone/s/recommended-repositories.

If there are ethical or legal restrictions on sharing a de-identified data set, please explain them in detail (e.g., data contain potentially sensitive information, data are owned by a third-party organization, etc.) and who has imposed them (e.g., an ethics committee). Please also provide contact information for a data access committee, ethics committee, or other institutional body to which data requests may be sent. If data are owned by a third party, please indicate how others may request data access.

If the reviewer comments include a recommendation to cite specific previously published works, please review and evaluate these publications to determine whether they are relevant and should be cited. There is no requirement to cite these works unless the editor has indicated otherwise.

Additional Editor Comments:

On the basis of the consistent comments received from

4 independent reviewers, the manuscript requires malore revisione and improvements before being considered further for publication in Plos One.

[Note: HTML markup is below. Please do not edit.]

Reviewers' comments:

Reviewer's Responses to Questions

**Comments to the Author**

1. Is the manuscript technically sound, and do the data support the conclusions?

The manuscript must describe a technically sound piece of scientific research with data that supports the conclusions. Experiments must have been conducted rigorously, with appropriate controls, replication, and sample sizes. The conclusions must be drawn appropriately based on the data presented.

Reviewer #1: Yes

Reviewer #2: Partly

Reviewer #3: Partly

Reviewer #4: Yes

**********

2. Has the statistical analysis been performed appropriately and rigorously?

Reviewer #1: N/A

Reviewer #2: Yes

Reviewer #3: Yes

Reviewer #4: Yes

**********

3. Have the authors made all data underlying the findings in their manuscript fully available?

The PLOS Data policy requires authors to make all data underlying the findings described in their manuscript fully available without restriction, with rare exception (please refer to the Data Availability Statement in the manuscript PDF file). The data should be provided as part of the manuscript or its supporting information, or deposited to a public repository. For example, in addition to summary statistics, the data points behind means, medians and variance measures should be available. If there are restrictions on publicly sharing data—e.g. participant privacy or use of data from a third party—those must be specified.

Reviewer #1: Yes

Reviewer #2: Yes

Reviewer #3: Yes

Reviewer #4: Yes

**********

4. Is the manuscript presented in an intelligible fashion and written in standard English?

PLOS ONE does not copyedit accepted manuscripts, so the language in submitted articles must be clear, correct, and unambiguous. Any typographical or grammatical errors should be corrected at revision, so please note any specific errors here.

Reviewer #1: Yes

Reviewer #2: Yes

Reviewer #3: Yes

Reviewer #4: Yes

**********

5. Review Comments to the Author

Please use the space provided to explain your answers to the questions above. You may also include additional comments for the author, including concerns about dual publication, research ethics, or publication ethics. (Please upload your review as an attachment if it exceeds 20,000 characters)

Reviewer #1: 1.What is the background for exploring the impact of the combined action of BPEF and shear field? It should be introduced briefly in the abstract.

2.Was the O/W emulsion used as the research object?

3.In abstract and conclusion, some quantitative results must be given.

4.How did you distinguish the vectors and scalars?

5.For some equations used in this work, some references are necessary.

6.What is the value of interfacial tension for the oil and water?

7.A diagram of model should be given, including the scalar, boundary conditions for the shear and electric fields.

Reviewer #2: This research focuses on the numerical simulation of emulsion evolution under the coupled action of a bidirectional pulsed electric field (BPEF) and a shear field, aiming to explore synergistic demulsification mechanisms.The coupled BPEF-shear field demulsification mechanism is a meaningful extension of existing single-field (electric or shear) studies.The LBM-based model is well-established in multiphase flow simulation, and the parameter setting, boundary conditions, and validation (e.g., Dr correlation with average droplet radius) are logically consistent.However, several limitations and ambiguities need clarification for completeness and reproducibility.

1. In the LBM color model, the free parameters αk and Ak are critical for phase separation and interface stability. The manuscript only states αk ∈(0,1) but does not specify the exact values used in simulations. How were these parameters calibrated to ensure immiscibility between oil and water, and what sensitivity analysis was conducted to verify their impact on simulation results?

2. The Poisson equation for electric field calculation (Section 2.1) is partially truncated in the manuscript. Could the authors provide the complete equation and clarify the derivation of the electric field force term FE using the Maxwell stress tensor? Additionally, how was the charge density ρE determined in the emulsion system, and did it account for polarization-induced charges on oil-water interfaces?

3. The boundary conditions for the left/right boundaries are described as “rebound boundary conditions,” but Equations (31)-(33) appear redundant (e.g., f2 (0,y)=f4 (0,y) and f4 (0,y)=f2 (0,y)). Could the authors correct or clarify the boundary condition formulation, and explain how it ensures mass conservation and flow field continuity?

4. The manuscript neglects gravity and temperature effects (Section 3), stating they are “usually factors to be considered in reality.” Given that industrial emulsions are often affected by gravity-driven sedimentation, how do the authors justify this simplification? Is there any evidence that gravity would not significantly alter the observed synergistic demulsification trends?

5. The initial simulation setup includes 9100 oil droplets (1 grid unit diameter) occupying 10% of the flow field area. However, the manuscript does not report the droplet size distribution after aggregation or validate the simulation against experimental data (e.g., droplet coalescence rates, demulsification efficiency). Has the model been validated with experimental results from similar electric-shear coupling systems, and if not, what plans exist to confirm its predictive accuracy?

6. Turbulence is excluded from the model, but high shear rates (e.g.,γ=5×10−4) may induce turbulent flow in real systems. How was the laminar flow assumption justified for the selected shear rate range, and what is the maximum shear rate for which the model remains valid?

7. In Section 3.2.2, high electric field intensity (V=400) causes droplet breakage when the electric field is perpendicular to the shear direction, but not when parallel. The manuscript attributes this to "balanced forces," but no quantitative analysis of force components (electric field force, shear force, surface tension) is provided. Could the authors present a force balance model to mathematically explain why perpendicular directions lead to breakage at high intensities?

8. The quantitative analysis (Section 5) uses Dr=Rave /2 under the assumption of spherical droplets. However, the manuscript notes that droplets deform into elliptical or elongated shapes under electric/shear forces. How does droplet deformation affect the validity of the Dr-based aggregation degree characterization, and was a correction factor applied for non-spherical droplets?

9. In Figure 9, V=500 shows similar demulsification efficiency to V=100 when the electric field is parallel to the shear direction, attributed to "low-velocity areas" induced by high electric intensity. However, the streamline analysis (Section 4.2) does not explicitly visualize these low-velocity regions. Could the authors provide additional flow field velocity contour plots to confirm this phenomenon, and explain the mechanism by which high electric intensity generates such regions?

10. The manuscript uses "typical crude oil parameters" (Table 1), but crude oil properties (e.g., dielectric constant, viscosity) vary widely across oilfields. How generalizable is the proposed demulsification strategy to emulsions with different oil compositions or contaminants (e.g., surfactants)? Are there any parameter ranges for which the synergistic effect of BPEF and shear fields would be compromised?

Reviewer #3: This paper uses the lattice Boltzmann method (LBM) to simulate the demulsification process of an emulsion under the coupled effects of a bidirectional pulsed electric field (BPEF) and a shear field. However, before it can be considered for publication, some points need further clarification. Some specific comments are outlined below:

1. The use of a 2D D2Q9 model is not sufficiently justified. Droplet coalescence and breakup are inherently three-dimensional, and the manuscript does not discuss the errors introduced by reducing the problem to 2D.

2. The simulations proceed directly to emulsion evolution without numerical validation. No comparison with analytical theory or experimental data is provided, which weakens confidence in the results.

3. The metric Dr (area-to-perimeter ratio) mixes shape deformation and aggregation effects. Its physical meaning is ambiguous and may lead to misleading interpretation. Separate metrics should be used.

4. The discussion of flow fields is largely descriptive. The influence of the electric field is not explained from a force or stress balance perspective, especially for the inhibition of aggregation at high voltage.

Reviewer #4: This manuscript establishes an LBM-based coupled electric–shear model to study droplet coalescence under a bidirectional triangular pulsed electric field. The topic is relevant to demulsification, and the manuscript is generally well-structured with clear qualitative trends.

1.(Section 2 “Numerical method”, electrostatics part) Poisson equation uses ε_b(water permittivity), and Eqs. (1)–(3) define ∇^2 ϕ, E=-∇ϕ, and Maxwell-stress-based force. Clarify why the present Poisson formulation is appropriate for an oil–water two-phase system and how permittivity contrast is handled。

2. (Section 3 “Simulation results and discussion”, initial setup): 256×256 grid, 9100 droplets, droplet diameter = 1 grid unit, ~10% area fraction. Please include at least one resolution or initial droplet-size sensitivity test (e.g., droplet diameter higher grid resolution) to demonstrate that the main conclusions (coalescence rate/Dr trends) are not dominated by lattice-scale effects.

3. Nondimensionalization needs clearer physical interpretation。Section 3, Eqs. (38)–(41) give nondimensionalization of length/time/voltage and material properties. It is difficult to infer what physical magnitudes (electric field strength, shear rate) correspond to the chosen nondimensional settings and whether they match typical demulsification conditions.Please add a short explanation (or a small table) mapping dimensional to nondimensional quantities and provide the approximate physical orders of magnitude for electric field strength and shear rate.

4. Breakup and “periodic aggregation–breakage” require an objective quantitative criterion.Section 3.2.2 reports fragmentation at higher field intensity and mentions periodic aggregation–breakage. The discussion states breakup occurs when electric + shear effects exceed surface tension. The current presentation is mainly qualitative; a reproducible breakup criterion and quantification of “periodicity” are missing.

5. Table 1 reports “Dielectric constant” in F/m (oil: 0.3×10⁻⁹ F/m; water: 7.8×10⁻⁹ F/m). F/m corresponds to absolute permittivity, while “dielectric constant” is often interpreted as relative permittivity (dimensionless).Please use consistent terminology (permittivity vs dielectric constant) and ensure units match the intended quantity.

6. Fig. 8–9 quantitative discussion compares shear rates and highlights an “optimal” trend. Streamline analysis explains the flow-direction change lags behind electric-field switching due to inertia/viscosity. The rationale for chosen shear-rate points and their relation to T_0/switching frequency is not discussed.

**********

6. PLOS authors have the option to publish the peer review history of their article (what does this mean?). If published, this will include your full peer review and any attached files.

If you choose “no”, your identity will remain anonymous but your review may still be made public.

**Do you want your identity to be public for this peer review?**  For information about this choice, including consent withdrawal, please see our Privacy Policy.

Reviewer #1: No

Reviewer #2: No

Reviewer #3: No

Reviewer #4: No

**********

[NOTE: If reviewer comments were submitted as an attachment file, they will be attached to this email and accessible via the submission site. Please log into your account, locate the manuscript record, and check for the action link "View Attachments". If this link does not appear, there are no attachment files.]

To ensure your figures meet our technical requirements, please review our figure guidelines: https://journals.plos.org/plosone/s/figures

You may also use PLOS’s free figure tool, NAAS, to help you prepare publication quality figures: https://journals.plos.org/plosone/s/figures#loc-tools-for-figure-preparation.

NAAS will assess whether your figures meet our technical requirements by comparing each figure against our figure specifications.

Attachment Submitted filename: Reviewer comments.pdf

# Author comment: Author response to Decision Letter 1

**DOI:** 10.1371/journal.pone.0336345.r002

17 Mar 2026

Dear Editors and Reviewers,

We appreciate the opportunity to revise our manuscript titled " Simulation of emulsion evolution in shear flow field under the control of bidirectional pulsed electric field" and are grateful for the insightful comments provided by the reviewers. We have carefully addressed all the comments of reviewers and revised the manuscript accordingly. Attached, you will find: (1) a detailed response letter outlining the changes made in response to the reviewers' suggestions, (2) a revised version of our manuscript with tracked changes, and (3) a clean version of our revised manuscript, free of any tracked changes.

As requested, we have meticulously addressed all reviewer comments and comprehensively improved the manuscript accordingly. Specifically: (1) The manuscript has been reformatted strictly following template; (2) The funding information has been updated. All revisions are marked in red in the manuscript; (3) The funding information has been updated in the cover letter to align with the acknowledgment section. Please let us know if any further modifications are needed.

sincerely yours,

Heping Wang

Response to the reviewers' comments:

Reviewer #1:

- Question Q1. What is the background for exploring the impact of the combined action of BPEF and shear field? It should be introduced briefly in the abstract.

- Question Q2. Was the O/W emulsion used as the research object?

- Question Q3. In abstract and conclusion, some quantitative results must be given.

- Question Q4. How did you distinguish the vectors and scalars?

- Question Q5. For some equations used in this work, some references are necessary.

- Question Q6. What is the value of interfacial tension for the oil and water?

- Question Q7. A diagram of model should be given, including the scalar, boundary conditions for the shear and electric fields.

Response: Thank you very much for your time in reviewing our manuscript. We sincerely appreciate your constructive suggestions, which have helped us significantly improve the quality of our work. We are also grateful for the opportunity to revise the manuscript based on your feedback. In this revision, we have carefully addressed your concerns point-by-point. We hope that the revised manuscript now meets your expectations and the journal’s standards.

- Question Q1. What is the background for exploring the impact of the combined action of BPEF and shear field? It should be introduced briefly in the abstract.

Response: We agree. In the revised Abstract, we have added the background information. We explained that while pure shear fields rely on random collisions (inefficient for fine droplets) and continuous electric fields can cause short-circuits, the synergy of BPEF and shear fields provides both mechanical dynamics and polarization forces to break the interfacial film resistance more effectively.

Copy: Electrostatic demulsification is a widely used technique for treating high-water-cut emulsions (O/W type). However, in high-water-cut emulsions, continuous electric fields often lead to short-circuiting and chain formation. Meanwhile, pure shear fields rely on random collisions, which is inefficient for fine droplets.

- Question Q2. Was the O/W emulsion used as the research object?

Response: Yes, the research object in this simulation is an O/W (Oil-in-Water) emulsion. We have explicitly stated this in the revised Abstract. The simulation generates oil droplets (dispersed phase) in a continuous water phase, with an oil concentration of approximately 10%.Revision: See Abstract (Line 2): "...treating high-water-cut emulsions (O/W type)." and Section 3 (Paragraph 1):This paper employs a computational grid of 256×256 units to simulate an oil-in-water (O/W) emulsion,

- Question Q3.In abstract and conclusion, some quantitative results must be given.

a)Response: We have included specific quantitative data in the revised manuscript to demonstrate the efficiency of the proposed method.In Abstract: "Under the optimal electric potential of V=300 and shear rate of, the average aggregation degree () reached 11.5, which represents a 4.7-fold increase compared to the pure shear field () and a 109% improvement compared to the low-voltage condition (V=100)."

In conclusion: “This represents a 4.7-fold increase compared to the pure shear field () and a 109% improvement relative to the low-voltage condition (V=100), demonstrating a superior balance between rapid droplet coalescence and stable morphological evolution.”

- Question Q4.How did you distinguish the vectors and scalars?

Response: We have added a "Symbol" table before the Introduction to clearly define the physical meanings and types of all variables.

Symbol Physical meaning Type

Density Scalar

Electric potential Scalar

Permittivity Scalar

Interfacial tension Scalar

Dynamic viscosity Scalar

Shear rate Scalar

Dimensionless voltage Scalar

Aggregation degree Scalar

Droplet area Scalar

Droplet perimeter Scalar

Time Scalar

Velocity Vector

Electric field Vector

Total body force Vector

Electric field force Vector

Surface tension force Vector

Gradient operator Vector operator

- Question Q5.For some equations used in this work, some references are necessary.

Response:We have added the necessary citations for the governing equations, including the Poisson equation for heterogeneous media, the Maxwell stress tensor, and the fundamental Lattice Boltzmann (LBM) evolution equations (e.g., [29] for the BGK model, [30] for the color model).

Copy: Due to the periodic change of the electric field, the electromagnetic induction effect will produce a magnetic field. However, in this paper, the current generated by the internal charge flow of the fluid is very small, so the influence of the magnetic field force is ignored. For the calculation of electric field distribution in the flow field, the central difference method is used in this paper. The electric potential distribution within the computational domain is governed by the Poisson equation for a heterogeneous dielectric medium [29]

Therefore, the LBM control equation in the form of BGK can be obtained [30]

[29] Jin, J. M. (2015). The finite element method in electromagnetics. John Wiley & Sons.

[30] Qian, Y. H., d'Humières, D., & Lallemand, P. (1992). Lattice BGK models for Navier-Stokes equation. Europhysics letters, 17(6), 479.

- Question Q6.What is the value of interfacial tension for the oil and water?

Response: We thank the reviewer for this question. In our simulation, the interfacial tension () between the oil and water phases is set to 0.02 (in lattice units). This parameter is a key factor in the color-gradient Lattice Boltzmann Method (LBM) used in this study, as it determines the magnitude of the surface tension force acting on the oil-water interface.

- Question Q7.A diagram of model should be given, including the scalar, boundary conditions for the shear and electric fields.

Response: We totally agree with the reviewer’s suggestion. We have added a comprehensive model schematic in the revised manuscript (see Fig. 1). This figure clearly illustrates:

The shear field boundary conditions: Represented by the moving walls with velocities and.

The electric field scalar boundary conditions: Defined by the applied potential and the ground.

The two study cases: Parallel and perpendicular configurations of the electric field relative to the shear flow.

This schematic provides a clear physical framework for the subsequent simulation results and analysis.

Fig. 1

Reviewer #2:

- Question Q1. In the LBM color model, the free parameters αk and Ak are critical for phase separation and interface stability. The manuscript only states αk ∈ (0,1) but does not specify the exact values used in simulations. How were these parameters calibrated to ensure immiscibility between oil and water, and what sensitivity analysis was conducted to verify their impact on simulation results?

- Question Q2. The Poisson equation for electric field calculation (Section 2.1) is partially truncated in the manuscript. Could the authors provide the complete equation and clarify the derivation of the electric field force term FE using the Maxwell stress tensor? Additionally, how was the charge density ρE determined in the emulsion system, and did it account for polarization-induced charges on oil-water interfaces?

- Question Q1.3. The boundary conditions for the left/right boundaries are described as "rebound boundary conditions," but Equations (31)-(33) appear redundant (e.g., f2 (0,y)=f4 (0,y) and f4 (0,y)=f2 (0,y)). Could the authors correct or clarify the boundary condition formulation, and explain how it ensures mass conservation and flow field continuity?

- Question Q4. The manuscript neglects gravity and temperature effects (Section 3), stating they are "usually factors to be considered in reality." Given that industrial emulsions are often affected by gravity-driven sedimentation, how do the authors justify this simplification? Is there any evidence that gravity would not significantly alter the observed synergistic demulsification trends?

- Question Q5. The initial simulation setup includes 9100 oil droplets (1 grid unit diameter) occupying 10% of the flow field area. However, the manuscript does not report the droplet size distribution after aggregation or validate the simulation against experimental data (e.g., droplet coalescence rates, demulsification efficiency). Has the model been validated with experimental results from similar electric-shear coupling systems, and if not, what plans exist to confirm its predictive accuracy?

- Question Q6. Turbulence is excluded from the model, but high shear rates (e.g.,γ=5×10−4) may induce turbulent flow in real systems. How was the laminar flow assumption justified for the selected shear rate range, and what is the maximum shear rate for which the model remains valid?

- Question Q7. In Section 3.2.2, high electric field intensity (V=400) causes droplet breakage when the electric field is perpendicular to the shear direction, but not when parallel. The manuscript attributes this to "balanced forces," but no quantitative analysis of force components (electric field force, shear force, surface tension) is provided. Could the authors present a force balance model to mathematically explain why perpendicular directions lead to breakage at high intensities?

- Question Q8. The quantitative analysis (Section 5) uses Dr=Rave /2 under the assumption of spherical droplets. However, the manuscript notes that droplets deform into elliptical or elongated shapes under electric/shear forces. How does droplet deformation affect the validity of the Dr-based aggregation degree characterization, and was a correction factor applied for non-spherical droplets?

- Question Q9. In Figure 9, V=500 shows similar demulsification efficiency to V=100 when the electric field is parallel to the shear direction, attributed to "low-velocity areas" induced by high electric intensity. However, the streamline analysis (Section 4.2) does not explicitly visualize these low-velocity regions. Could the authors provide additional flow field velocity contour plots to confirm this phenomenon, and explain the mechanism by which high electric intensity generates such regions?

- Question Q10. The manuscript uses "typical crude oil parameters" (Table 1), but crude oil properties (e.g., dielectric constant, viscosity) vary widely across oilfields. How generalizable is the proposed demulsification strategy to emulsions with different oil compositions or contaminants (e.g., surfactants)? Are there any parameter ranges for which the synergistic effect of BPEF and shear fields would be compromised?

Response: We would like to express our deepest gratitude for your thorough review and thoughtful suggestions on our manuscript. Your expertise has been valuable in guiding us to refine our research, and we truly appreciate the chance to submit a revised version. We have meticulously responded to every point you raised, and all corresponding revisions in the manuscript are marked in red. Once again, thank you for your time and support. We hope the revised version aligns with your expectations.

- Question Q1. In the LBM color model, the free parameters αk and Ak are critical for phase separation and interface stability. The manuscript only states αk ∈(0,1) but does not specify the exact values used in simulations. How were these parameters calibrated to ensure immiscibility between oil and water, and what sensitivity analysis was conducted to verify their impact on simulation results?

Response: In our simulations, the segregation parameter was set to to maintain a sharp interface while minimizing spurious currents. The interfacial tension was explicitly set to (variable surface Tension) using the continuum surface force method, ensuring precise control over the immiscibility. While a full sensitivity analysis was not included in the original manuscript, typical variations of in the range of [0.5, 0.9] are known to have negligible effects on macroscopic droplet dynamics in this model.

Copy:

To ensure phase separation and immiscibility, the segregation parameter is set to 0.7 in the recoloring step. The interfacial tension is controlled by the parameter , which is calibrated according to the desired macroscopic surface tension via the Laplace law.

- Question Q2. The Poisson equation for electric field calculation (Section 2.1) is partially truncated in the manuscript. Could the authors provide the complete equation and clarify the derivation of the electric field force term FE using the Maxwell stress tensor? Additionally, how was the charge density ρE determined in the emulsion system, and did it account for polarization-induced charges on oil-water interfaces?

Response: 1) Poisson equation: We apologize for the truncation in Eq. (1). The complete Poisson equation relating the electric potential to the free charge density in a heterogeneous medium is:

In our Leaky Dielectric Model (LDM) implementation, the potential distribution is primarily governed by the current conservation equation (assuming steady state), and the charge density is then derived from the Poisson equation.

2). On the electric force derivation: The electric force density is derived from the divergence of the Maxwell stress tensor .

By expand and using the vector identity,along with, we obtain: This formula explicitly includes the Coulomb force acting on free charges and the dielectric force acting on the interface.

3). On charge density and polarization: The charge density is determined dynamically in the simulation using Gauss's law:. It arises naturally at the interface due to the mismatch in electrical properties (conductivity and permittivity) between the oil and water phases (Maxwell-Wagner polarization). Furthermore, the term in our force equation explicitly accounts for the force resulting from the polarization of the dielectric interface.

Copy:The electric potential distribution within the computational domain is governed by the Poisson equation for a heterogeneous dielectric medium[29]:

(1)

- Question Q3. The boundary conditions for the left/right boundaries are described as "rebound boundary conditions," but Equations (31)-(33) appear redundant (e.g., f2 (0,y)=f4 (0,y) and f4 (0, y) =f2 (0, y)). Could the authors correct or clarify the boundary condition formulation, and explain how it ensures mass conservation and flow field continuity?

Response: We thank the reviewer for pointing out this inconsistency. We apologize for the confusion caused by an editing error in the manuscript.

Clarification on Boundary Conditions: In our simulation of infinite shear flow, the left and right boundaries are actually subject to Periodic Boundary Conditions, not rebound boundary conditions. The description of 'rebound boundary conditions' and the associated Equations (31)-(33) were remnants from a previous template and were included in error. We have removed them in the revised manuscript.

Correct Implementation: The correct boundary condition is implemented as: for all lattice directions .

Mass Conservation and Continuity: By using periodic boundary conditions, any fluid particle leaving the domain t

Attachment Submitted filename: Response to reviewers.doc

# Peer review: Decision Letter 1

**DOI:** 10.1371/journal.pone.0336345.r003

**Authors:** P. Davide Cozzoli, P. Davide Cozzoli

30 Mar 2026

PONE-D-25-56801R1

Simulation of emulsion evolution in shear flow field under the control of bidirectional pulsed electric field

PLOS One

Dear Dr. Wang,

Thank you for submitting your manuscript to PLOS ONE. After careful consideration, we feel that it has been revised appropriately, but does not fully meet PLOS ONE’s publication criteria as it currently stands. Therefore, we invite you to submit a revised version of the manuscript that addresses the points raised during the review process by reviewer #2.

==============================

**ACADEMIC EDITOR: Authors are requested to address the minor issues raised by reviewer #2.**

==============================

Please submit your revised manuscript by May 14 2026 11:59PM. If you will need more time than this to complete your revisions, please reply to this message or contact the journal office at plosone@plos.org. When you're ready to submit your revision, log on to https://www.editorialmanager.com/pone/ and select the 'Submissions Needing Revision' folder to locate your manuscript file.

Please include the following items when submitting your revised manuscript:

- A letter that responds to each point raised by the academic editor and reviewer(s). You should upload this letter as a separate file labeled 'Response to Reviewers'.
- A marked-up copy of your manuscript that highlights changes made to the original version. You should upload this as a separate file labeled 'Revised Manuscript with Track Changes'.
- An unmarked version of your revised paper without tracked changes. You should upload this as a separate file labeled 'Manuscript'.

If you would like to make changes to your financial disclosure, please include your updated statement in your cover letter. Guidelines for resubmitting your figure files are available below the reviewer comments at the end of this letter.

If applicable, we recommend that you deposit your laboratory protocols in protocols.io to enhance the reproducibility of your results. Protocols.io assigns your protocol its own identifier (DOI) so that it can be cited independently in the future. For instructions see: https://journals.plos.org/plosone/s/submission-guidelines#loc-laboratory-protocols. Additionally, PLOS ONE offers an option for publishing peer-reviewed Lab Protocol articles, which describe protocols hosted on protocols.io. Read more information on sharing protocols at https://plos.org/protocols?utm_medium=editorial-email&utm_source=authorletters&utm_campaign=protocols.

As the corresponding author, your ORCID iD is verified in the submission system and will appear in the published article. PLOS supports the use of ORCID, and we encourage all coauthors to register for an ORCID iD and use it as well. Please encourage your coauthors to verify their ORCID iD within the submission system before final acceptance, as unverified ORCID iDs will not appear in the published article. *Only*  the individual author can complete the verification step; PLOS staff *cannot*  verify ORCID iDs on behalf of authors.

We look forward to receiving your revised manuscript.

Kind regards,

P. Davide Cozzoli

Academic Editor

PLOS One

Journal Requirements:

If the reviewer comments include a recommendation to cite specific previously published works, please review and evaluate these publications to determine whether they are relevant and should be cited. There is no requirement to cite these works unless the editor has indicated otherwise.

Please review your reference list to ensure that it is complete and correct. If you have cited papers that have been retracted, please include the rationale for doing so in the manuscript text, or remove these references and replace them with relevant current references. Any changes to the reference list should be mentioned in the rebuttal letter that accompanies your revised manuscript. If you need to cite a retracted article, indicate the article’s retracted status in the References list and also include a citation and full reference for the retraction notice.

Additional Editor Comments:

The original reviewers are generally satisfied with the revisions made by the authors. Yet, reviewer #2 is requesting additional minor changes and improvements before the manuscript is accepted for publication.

[Note: HTML markup is below. Please do not edit.]

Reviewers' comments:

Reviewer's Responses to Questions

**Comments to the Author**

1. If the authors have adequately addressed your comments raised in a previous round of review and you feel that this manuscript is now acceptable for publication, you may indicate that here to bypass the “Comments to the Author” section, enter your conflict of interest statement in the “Confidential to Editor” section, and submit your "Accept" recommendation.

Reviewer #1: (No Response)

Reviewer #2: All comments have been addressed

Reviewer #3: All comments have been addressed

**********

2. Is the manuscript technically sound, and do the data support the conclusions?

The manuscript must describe a technically sound piece of scientific research with data that supports the conclusions. Experiments must have been conducted rigorously, with appropriate controls, replication, and sample sizes. The conclusions must be drawn appropriately based on the data presented.

Reviewer #1: (No Response)

Reviewer #2: Partly

Reviewer #3: Yes

**********

3. Has the statistical analysis been performed appropriately and rigorously?

Reviewer #1: (No Response)

Reviewer #2: Yes

Reviewer #3: Yes

**********

4. Have the authors made all data underlying the findings in their manuscript fully available?

The PLOS Data policy requires authors to make all data underlying the findings described in their manuscript fully available without restriction, with rare exception (please refer to the Data Availability Statement in the manuscript PDF file). The data should be provided as part of the manuscript or its supporting information, or deposited to a public repository. For example, in addition to summary statistics, the data points behind means, medians and variance measures should be available. If there are restrictions on publicly sharing data—e.g. participant privacy or use of data from a third party—those must be specified.

Reviewer #1: (No Response)

Reviewer #2: Yes

Reviewer #3: Yes

**********

5. Is the manuscript presented in an intelligible fashion and written in standard English?

PLOS ONE does not copyedit accepted manuscripts, so the language in submitted articles must be clear, correct, and unambiguous. Any typographical or grammatical errors should be corrected at revision, so please note any specific errors here.

Reviewer #1: (No Response)

Reviewer #2: Yes

Reviewer #3: Yes

**********

6. Review Comments to the Author

Please use the space provided to explain your answers to the questions above. You may also include additional comments for the author, including concerns about dual publication, research ethics, or publication ethics. (Please upload your review as an attachment if it exceeds 20,000 characters)

Reviewer #1: (No Response)

Reviewer #2: 1. Acronyms such as LBM,BGK which appear for the first time in the text, must be defined.

2、“ some scholars have found that changing the dielectric constant and conductivity can regulate droplet deformation in shear flow fields” The first word at the beginning of a sentence should be capitalised.

3、The variables in the equations in Section 2.1 and subsequent sections are not accompanied by units.

4、"the selected shear rate (≈ 280 s-1)�while the electric field strength (≈ 3 kV/cm) " Why not provide accurate figures?

5、“ In reality, gravity and temperature are usually factors to be considered, which are ignored in this paper.” Please provide the basis for this assumption and explain it.

6、“ In addition, the model in this paper cannot deal with the case of turbulence.” Could the author please explain whether the model studied in this paper is suited to laminar or turbulent flow conditions? If it is not suited to turbulent conditions, but the flow pattern of the emulsion becomes turbulent when the shear rate is too high, how should this be addressed?

Reviewer #3: The authors have addressed all my questions. The current manuscript is recommended to be accepted in PLOS One.

**********

7. PLOS authors have the option to publish the peer review history of their article (what does this mean?). If published, this will include your full peer review and any attached files.

If you choose “no”, your identity will remain anonymous but your review may still be made public.

**Do you want your identity to be public for this peer review?**  For information about this choice, including consent withdrawal, please see our Privacy Policy.

Reviewer #1: No

Reviewer #2: No

Reviewer #3: No

**********

[NOTE: If reviewer comments were submitted as an attachment file, they will be attached to this email and accessible via the submission site. Please log into your account, locate the manuscript record, and check for the action link "View Attachments". If this link does not appear, there are no attachment files.]

To ensure your figures meet our technical requirements, please review our figure guidelines: https://journals.plos.org/plosone/s/figures

You may also use PLOS’s free figure tool, NAAS, to help you prepare publication quality figures: https://journals.plos.org/plosone/s/figures#loc-tools-for-figure-preparation.

NAAS will assess whether your figures meet our technical requirements by comparing each figure against our figure specifications.

# Author comment: Author response to Decision Letter 2

**DOI:** 10.1371/journal.pone.0336345.r004

13 May 2026

Dear Editors and Reviewers:

We appreciate the opportunity to revise our manuscript titled " Simulation of emulsion evolution in shear flow field under the control of bidirectional pulsed electric field" and are grateful for the insightful comments provided by the reviewers. We have carefully addressed all the comments of reviewers and revised the manuscript accordingly.

- Question Q1: Acronyms such as LBM, BGK which appear for the first time in the text, must be defined.

Response: Thank you for this valuable comment. We agree that all acronyms should be clearly defined at their first occurrence.

In the revised manuscript, we have carefully checked and corrected this issue. Specifically: “LBM” has been revised to “lattice Boltzmann method (LBM)” at its first appearance in Introduction.

Copy: “BGK” has been revised to “Bhatnagar–Gross–Krook (BGK)” when first introduced in the collision model description.

All subsequent occurrences use the abbreviations consistently.

- Question Q2: “some scholars have found that changing the dielectric constant and conductivity can regulate droplet deformation in shear flow fields” The first word at the beginning of a sentence should be capitalised.

Response: Thank you for pointing out this grammatical issue.

We have corrected the sentence in the revised manuscript by capitalizing the first word. Revised as: “Some scholars have found that changing the dielectric constant and conductivity can regulate droplet deformation in shear flow fields.” In addition, we have conducted a thorough proofreading of the entire manuscript to ensure that all sentences begin with proper capitalization.

- Question Q3: The variables in the equations in Section 2.1 and subsequent sections are not accompanied by units.

Response: Thank you for this important comment.

We would like to clarify that the governing equations in this study are primarily expressed in a dimensionless lattice Boltzmann framework, where variables are normalized using lattice units (lu) and time steps (ts). Therefore, most variables in Section 2.1 do not carry conventional SI units.

To address this concern and improve clarity, we have made the following revisions:

Supplemented Table 3, which provides: Mapping between dimensionless parameters and their corresponding physical magnitudes and units (e.g., shear rate, electric field strength).

Improved the Symbol Table, where:

Units (SI) are now explicitly listed for each physical variable where applicable.

- Question Q4: the selected shear rate (≈ 280 s-1)�while the electric field strength (≈ 3 kV/cm) " Why not provide accurate figures?

Response: Thank you for this insightful comment.

The reported values (≈ 280 s⁻¹ and ≈ 3 kV/cm) are obtained through dimensional mapping between lattice Boltzmann units and physical quantities based on characteristic scaling (see Table 3). Due to the inherent scaling freedom of the lattice Boltzmann framework, this mapping is not unique, and therefore these values represent order-of-magnitude estimates rather than exact physical quantities.

To clarify this point, we have revised the manuscript as follows: The description in the main text has been modified to explicitly state that these values correspond to typical industrial ranges rather than precise measurements.

Table 3 has been updated to clarify that the reported values are approximate and scaling-dependent.

A note has been added to explain the origin and limitation of the dimensional conversion.

These revisions improve the physical interpretation and transparency of the simulation parameters.

Question5: In reality, gravity and temperature are usually factors to be considered, which are ignored in this paper. Please provide the basis for this assumption and explain it.

Response: We appreciate the reviewer’s valuable comment regarding the physical completeness of the model. In this study, we intentionally adopted an isothermal assumption and neglected gravity to isolate and clarify the synergistic mechanisms of the Bidirectional Pulse Electric Field (BPEF) and the shear field. The justifications for these assumptions are as follows:

1. Justification for Neglecting Gravity (Bond Number Analysis):

The relative importance of gravity versus interfacial tension can be characterized by the dimensionless Bond number ():

where is the density difference, is gravity, is the droplet radius, and is the interfacial tension.

In typical micro-scale emulsions (droplet size), the surface tension force dominates over the gravitational/buoyancy force. For the parameters used in our study, the Bond number is much smaller than unity (), indicating that gravity has a negligible effect on droplet deformation and short-range dipole-dipole interactions compared to Maxwell stress and capillary stress.

Therefore, neglecting gravity is a standard and justified simplification in studies focusing on the electro-hydrodynamics (EHD) of fine emulsions.

2. Justification for the Isothermal Assumption:

Mechanism Isolation: This work focuses on the mechanical and electrical coupling (the competition between Maxwell stress, viscous stress, and surface tension). By maintaining a constant temperature (isothermal condition), we can isolate the effects of electric field frequency and phase without the interference of thermocapillary (Marangoni) flows or temperature-dependent viscosity variations.

Time Scale: The characteristic time for droplet collision and coalescence under strong electric and shear fields is significantly shorter than the time scale of significant heat transfer or temperature-induced property changes in the bulk phase.

Practicality in LBM: In most Lattice Boltzmann Method (LBM) studies of electro-demulsification, the isothermal assumption is a standard benchmark to establish the fundamental control laws before introducing complex multi-physics thermal coupling.

Copy: To isolate the synergistic mechanism of the bidirectional pulsed electric field (BPEF) and the shear field, this study assumes an isothermal environment and neglects the effect of gravity.

For micro-scale emulsions (with typical droplet sizes of ), the relative importance of gravity compared to interfacial tension is characterized by the Bond number (). Given the micro-scale dimensions and fluid properties, the Bond number is much smaller than unity (), indicating that the droplet dynamics are completely dominated by the Maxwell stress, viscous shear, and interfacial capillary forces, making gravity negligible.

Furthermore, the isothermal assumption is adopted to deliberately exclude the complex interference of thermocapillary (Marangoni) flows and temperature-dependent viscosity variations, thereby allowing a focused investigation into the pure electro-hydrodynamic (EHD) coupling laws.

- Question 6: In addition, the model in this paper cannot deal with the case of turbulence.” Could the author please explain whether the model studied in this paper is suited to laminar or turbulent flow conditions? If it is not suited to turbulent conditions, but the flow pattern of the emulsion becomes turbulent when the shear rate is too high, how should this be addressed?

Response: We thank the reviewer for pointing out this boundary condition. The current LBM-EHD model is indeed a laminar flow solver. We believe this is the appropriate regime for the physical scale and the scientific objectives of this study for the following reasons:

1. Reynolds Number () at the Micro-scale:

In our study, the characteristic length scale is the droplet diameter () and the gap between electrodes is small. The Reynolds number () remains low even at relatively high shear rates because of the small characteristic length () and the high viscosity () of the oil phase. In laboratory-scale electrostatic coalescers and microfluidic devices, the flow typically remains in the laminar or transitional regime rather than fully developed turbulence.

2. Local Laminar Assumption (Kolmogorov Scale):

Even if the global flow in an industrial-scale demulsifier were turbulent, the interaction between two microscopic droplets often occurs at scales smaller than the Kolmogorov microscale (the smallest dissipative scales of turbulence). At this sub-grid scale, the local relative velocity field can be effectively approximated as a locally linear laminar shear flow. Therefore, our model provides a high-fidelity representation of the "local" physics of coalescence that occurs within a larger turbulent system.

3. Addressing High-Shear Turbulence:

If the shear rate were increased to a point where the flow becomes inherently turbulent, the current model would need to be addressed in the following ways:

Numerical Adjustment: We would need to incorporate a Sub-Grid Scale (SGS) model, such as Large Eddy Simulation (LES) within the LBM framework, to account for turbulent eddy viscosity and energy dissipation.

Physical Effect: In a turbulent regime, the random fluctuations would likely overcome the structured dipole-dipole attraction of the BPEF. The "synergistic effect" we identified might weaken as the deterministic shear trajectory is replaced by stochastic turbulent collisions.

Copy: "In addition the current model assumes a laminar regime, which is consistent with the micro-scale droplet interactions studied. For industrial applications involving high-Reynolds-number turbulence, the model would require integration with Large Eddy Simulation (LES) methods to account for stochastic turbulent fluctuations."

In addition, we have included streamlines of the flow field at two higher shear rates (Figure X: 0.002 and 0.003). As shown, the flow exhibits regions of accelerated and decelerated motion and localized vortices under high shear, but no fully developed turbulence is observed. This indicates that the flow remains predominantly laminar, albeit with strong shear gradients.

It should be noted that the present lattice Boltzmann model does not incorporate turbulence modeling (e.g., LES or RANS), and therefore cannot capture turbulence-induced multi-scale droplet breakup. Consequently, the model is strictly applicable to laminar flow regimes. For flows exceeding the laminar regime, advanced LBM approaches with turbulence closure, such as LES- or MRT-based schemes, would be required.

\

Streamline diagram of the flow field at a shear rate of 0.002

Streamline diagram of the flow field at a shear rate of 0.003

Attachment Submitted filename: response letter.docx

# Editor report: Decision Letter 2

**DOI:** 10.1371/journal.pone.0336345.r005

**Authors:** P. Davide Cozzoli, P. Davide Cozzoli, P. Davide Cozzoli

19 May 2026

Simulation of emulsion evolution in shear flow field under the control of bidirectional pulsed electric field

PONE-D-25-56801R2

Dear Dr. Wang,

We’re pleased to inform you that your manuscript has been judged scientifically suitable for publication and will be formally accepted for publication once it meets all outstanding technical requirements.

Within one week, you’ll receive an e-mail detailing the required amendments. When these have been addressed, you’ll receive a formal acceptance letter and your manuscript will be scheduled for publication.

An invoice will be generated when your article is formally accepted. Please note, if your institution has a publishing partnership with PLOS and your article meets the relevant criteria, all or part of your publication costs will be covered. Please make sure your user information is up-to-date by logging into Editorial Manager at Editorial Manager® and clicking the ‘Update My Information' link at the top of the page. For questions related to billing, please contact billing support.

If your institution or institutions have a press office, please notify them about your upcoming paper to help maximize its impact. If they’ll be preparing press materials, please inform our press team as soon as possible -- no later than 48 hours after receiving the formal acceptance. Your manuscript will remain under strict press embargo until 2 pm Eastern Time on the date of publication. For more information, please contact onepress@plos.org.

Kind regards,

P. Davide Cozzoli

Academic Editor

PLOS One

Additional Editor Comments (optional):

Reviewers' comments:

# Editor report: Acceptance letter

**DOI:** 10.1371/journal.pone.0336345.r006

**Authors:** P. Davide Cozzoli, P. Davide Cozzoli, P. Davide Cozzoli

PONE-D-25-56801R2

PLOS One

Dear Dr. Wang,

I'm pleased to inform you that your manuscript has been deemed suitable for publication in PLOS One. Congratulations! Your manuscript is now being handed over to our production team.

At this stage, our production department will prepare your paper for publication. This includes ensuring the following:

* All references, tables, and figures are properly cited

* All relevant supporting information is included in the manuscript submission,

* There are no issues that prevent the paper from being properly typeset

You will receive further instructions from the production team, including instructions on how to review your proof when it is ready. Please keep in mind that we are working through a large volume of accepted articles, so please give us a few days to review your paper and let you know the next and final steps.

Lastly, if your institution or institutions have a press office, please let them know about your upcoming paper now to help maximize its impact. If they'll be preparing press materials, please inform our press team within the next 48 hours. Your manuscript will remain under strict press embargo until 2 pm Eastern Time on the date of publication. For more information, please contact onepress@plos.org.

You will receive an invoice from PLOS for your publication fee after your manuscript has reached the completed accept phase. If you receive an email requesting payment before acceptance or for any other service, this may be a phishing scheme. Learn how to identify phishing emails and protect your accounts at https://explore.plos.org/phishing.

If we can help with anything else, please email us at customercare@plos.org.

Thank you for submitting your work to PLOS ONE and supporting open access.

Kind regards,

PLOS ONE Editorial Office Staff

on behalf of

Dr. P. Davide Cozzoli

Academic Editor

PLOS One
